# Meta-Analysis of the Validity of General Mental Ability for Five Performance Criteria: Hunter and Hunter (1984) Revisited

**DOI:** 10.3389/fpsyg.2019.02227

**Published:** 2019-10-17

**Authors:** Jesús F. Salgado, Silvia Moscoso

**Affiliations:** Faculty of Labor Relations, University of Santiago de Compostela, Santiago de Compostela, Spain

**Keywords:** general mental ability, meta-analysis, job performance ratings, grades, production records, instructor ratings, work sample test, training

## Abstract

This paper presents a series of meta-analyses of the validity of general mental ability (GMA) for predicting five occupational criteria, including supervisory ratings of job performance, production records, work sample tests, instructor ratings, and grades. The meta-analyses were conducted with a large database of 467 technical reports of the validity of the General Aptitude Test Battery (GATB) which included 630 independent samples. GMA showed to be a consistent predictor of the five criteria, but the magnitude of the operational validity was not the same across the five criteria. Results also showed that job complexity is a moderator of the GMA validity for the performance criteria. We also found that the GMA validity estimates are slightly smaller than the previous ones obtained by Hunter and Hunter (1984). Finally, we discuss the implications of these findings for the research and practice of personnel selection.

## Introduction

General mental ability (GMA) tests are one of the most valid construct-based predictors of job performance and training success. Thousands of validity studies and many meta-analyses have shown that they are excellent predictors of different organizational criteria, such as supervisory ratings, work sample tests, job knowledge acquisition, grades, production records, instructor ratings, promotions, sales, and wages, and that the correlation between GMA and performance appears to be similar across jobs that differ considerably in content (Schmidt and Hunter, 1998; Murphy, 2002; Ones et al., 2012; Schmitt, 2014; Salgado, 2017a; Berges et al., 2018; Rodríguez and López-Basterra, 2018). Furthermore, the theoretical foundations of GMA are stronger than for any other personnel selection measure (Schmidt and Hunter, 1998; Hunt, 2011; Deary, 2012; Salgado, 2017a). For all these reasons, researchers have claimed that GMA tests play a fundamental role in personnel selection (Schmidt and Hunter, 1998; Murphy, 2002; Schmitt, 2014). Scherbaum et al. (2012) have argued that GMA is now more important than ever because of the changing nature of work environments.

Among the research work that has examined the validity of GMA, the meta-analyses of John E. Hunter occupy a special place. These meta-analyses were based on essentially the same dataset and the results were reported in several publications (Hunter, 1980, 1983a, 1986; Hunter and Hunter, 1984), with little differences and additions. His most cited publication was the article published in Psychological Bulletin (Hunter and Hunter, 1984), but some informative pieces appear in other reports and articles (e.g., Hunter, 1983a, 1986). Hunter carried out the first meta-analysis that examined the validity generalization evidence of the General Aptitude Test Battery (GATB), and this meta-analytic effort was made using the largest existing database of primary studies for a single battery. The GATB has been now renamed as Ability Profiler, Forms 1 and 2 (Mellon et al., 1996). The GATB (and the Ability Profiler) consists of 12 tests that assess General Learning Ability (G) and eight specific abilities, including Verbal (V), Numerical (N), Spatial (S), Form Perception (P), Clerical Perception (Q), Motor Coordination (K), Finger Dexterity (F), and Manual Dexterity (M). Table 1 describes the tests included in the GATB. For example, the G composite is created by the sum of vocabulary (test 4), arithmetic reasoning (test 6), and three-dimensional space (test 3); Verbal aptitude (V) is assessed with test 4; Spatial aptitude (S) is evaluated with test 3, and Numerical aptitude (N) is evaluated with test 2 (computation) and test 6 (arithmetic reasoning). Table 2 shows the correlations among the abilities measured by the GATB. The raw test scores are converted into standardized scores with a mean of 100 and a standard deviation of 20 for each of the GATB abilities. Although the GATB included a GMA composite (i.e., G or General Learning Ability), Hunter (1983a, 1986; Hunter and Hunter, 1984) created a second GMA composite summing up G, V, and N, and called this composite as GVN.

**Table 1:** The nine aptitudes measured by the GATB and their respective tests.

**Aptitude**	**Tests**
G—General learning ability	Test 3—Three dimensional space
	Test 4—Vocabulary
	Test 6—Arithmetic reasoning
V—Verbal aptitude	Test 4—Vocabulary
N—Numerical aptitude	Test 2—Computation
	Test 6—Arithmetic reasoning
S—Spatial aptitude	Test 3—Three-dimensional space
P—Form perception	Test 5—Tool matching
	Test 7—Form matching
Q—Clerical perception	Test 1—Name comparison
K—Motor coordination	Test 8—Mark making
F—Finger dexterity	Test 11—Assemble
	Test 12—Disassemble
M—Manual dexterity	Part 9—Place
	Part 10—Turn

**Table 2:** Observed and corrected correlations among the GATB abilities.

	**G**	**V**	**N**	**S**
G	–	0.92	0.98	0.82
V	0.81	–	0.80	0.55
N	0.86	0.67	–	0.62
S	0.71	0.46	0.51	–
*r_*xx*_*	0.92	0.85	0.83	0.81

*Observed correlations were taken from U.S. Department of Labor (1970; section III, p. 34) for 23,428 workers. Observed correlations appear below the diagonal. Correlations corrected for measurement error in both abilities appear above the diagonal. The correlations were not corrected for range restriction*.

In order to conduct his meta-analyses and to correct the observed validities for criterion reliability and range restriction, Hunter (1980, Hunter, 1983a, 1986; Hunter and Hunter, 1984) assumed the values of 0.60 and 0.80 for the reliability of job proficiency and training, respectively. In addition, he empirically derived the distributions of range restriction based on the information provided by the validity studies. Both, the assumed reliability estimates were criticized by the NAS Panel (Hartigan and Wigdor, 1989) and the range restriction distributions were criticized on different reasons by Hartigan and Wigdor (1989) and Berry et al. (2014).

The meta-analyses of (Hunter, 1983a, 1986; Hunter and Hunter, 1984) made two main findings. First, the average operational validity of GMA was 0.45 for predicting job proficiency and 0.54 for predicting training success, and, although the validity of GMA varied across job families, it never approached zero. Second, job complexity moderated the validity of GMA, so that the validity increased as job complexity increased.

According to the Manual of the GATB (U.S. Department of Labor, 1970; section III, pp. 62–124), the abilities measured by the GATB were validated against objective criteria (e.g., production records, work sample tests, grades) and subjective criteria (e.g., supervisory ratings of job performance, and instructor rating of training success) for 446 occupations.

No meta-analyses tested the validity of the GMA, as assessed with the GATB, for the specific criteria, i.e., supervisory ratings, productions records, work sample tests, instructor ratings, and grades. Due to the differences among criteria, for instance, the differences in reliability, Tenopyr (2002) suggested that objective and subjective methods of job performance measurement should be considered separately in connection with the validity of GMA (see, for instance, AlDosiry et al., 2016, as an example of the use objective sales performance and subjective sales performance). It is important to remark that neither Hunter's meta-analyses nor other meta-analyses estimated empirically the reliability of the criteria used in the GATB studies, particularly, the interrater reliability of supervisory and instructor performance ratings. Moreover, there is another unexplored issue: the validity of the two alternative compounds of GMA mentioned above. Also, it remains unexamined whether job complexity moderates GMA validity similarly across the specific criteria.

As a whole, the purpose of this article is to shed further light on these five critical issues that the existing meta-analyses have overlooked: (a) the estimation of the validity of GMA for the five specific criteria; (b) the moderator role of job complexity on the validity of GMA for predicting the five criteria; (c) the comparison of the validity of the two GMA composites of the GATB (i.e., G and GVN); (d) the reliability of the criterion measures used in the GTAB validity studies, particularly, the interrater reliability of supervisory and instructor performance ratings; and (e) the empirical distributions of range restriction in the GATB validity studies.

## The Meta-analyses of John E. Hunter on the Validity of GMA

The meta-analyses that Hunter reported in several publications (Hunter, 1980, 1983a, 1986; Hunter and Hunter, 1984) were conducted with a database of 515 criterion validity studies, of which 425 used a measure of job proficiency, and 90 used a measure of training success. The total sample size was 38,620 individuals. For these reasons, and also due to its many methodological innovations (e.g., the manner of establishing the range restriction distributions; the job complexity classification based on Fine's functional job analysis; the construction of a new GMA composite by adding up G, V, and N scores), Hunter's findings have served for decades as the primary reference for the validity evidence of GMA as a predictor of job proficiency and training success, and the gold standard with which to compare other meta-analyses.

Hunter's database and meta-analyses were reviewed by a panel of the U.S. National Academy of Sciences (NAS) (Hartigan and Wigdor, 1989), who examined the evidence of validity generalization of the GATB. The findings of the NAS panel totally agreed with Hunter and Hunter (1984; see also, Hunter, 1983a) findings, concerning the observed validity estimates.

Recently, new methodological advances on indirect range restriction (IRR) correction in meta-analysis methods have been applied to Hunter and Hunter's (1984) estimates of the validity of the GMA. Schmidt and his colleagues (Hunter et al., 2006; Schmidt et al., 2006, 2008) have developed a new method to correct for IRR that can be applied when it is not possible to apply Thorndike's (1949) Case III formula because some information is lacking. When Schmidt et al. (2008) re-analyzed Hunter and Hunter's (1984) estimates with the new IRR formula, they found an increment of around 20% on average in the magnitude of the GMA validity. For example, while Hunter and Hunter (1984; see also Hunter, 1986) reported operational validity coefficients of 0.56, 0.50, and 0.39 for high, medium, and low complexity jobs, respectively, Schmidt et al. (2008) reported operational validity coefficients of 0.68, 0.62, and 0.50, respectively. Thus, these last results suggest that GMA tests can be even better predictors than Hunter and Hunter (1984) findings showed.

Nevertheless, as we posited above, several research issues remain unexplored, and also there are several characteristics of Hunter's meta-analytic distributions of artifacts that require a re-examination. In the next sections, we comment on these issues.

## Performance and Training Criteria

A first issue which has not been thoroughly examined relates to the specific criteria used in the validity studies. According to the Manual of the GATB (U.S. Department of Labor, 1970; section III, pp. 47–52), both objective and subjective criteria were used to validate the GATB. The objective criteria included production records, work sample tests, school grades, and grade point average (GPA). The subjective criteria consisted of supervisory ratings and instructor ratings. Therefore, job performance was assessed with three types of criteria, including production records, work sample tests, and supervisor ratings. For its part, training success was evaluated with grades and instructor ratings. As suggested by McDaniel et al. (1994), the criterion-type distinctions are relevant because operational validity typically varies by criterion type, and separate analyses are warranted because of the potential differences in average reliability for the various criteria types.

Concerning the validity of the GATB for predicting this variety of criteria, two points require more attention. First, different criteria can capture distinct aspects of job performance. A meta-analysis by Bommer et al. (1995) found that the overall correlations between ratings and alternative “objective” measures of performance were relatively low. For instance, consider work sample tests and supervisor ratings. In military databases, the GMA validities are higher for objective job sample measures of job performance than for supervisory ratings (Hunter and Hunter, 1984). This finding might indicate that GMA predicts better maximum performance (e.g., work samples tests) than typical performance (e.g., supervisory ratings, average production records). This fact is an important reason to consider the validity of GMA against these criteria separately. However, this issue was not examined by Hunter and Hunter (1984).

A second point is related to the reliability of the criteria. There has been some debate regarding the appropriateness of interrater reliability of supervisor ratings (Murphy and De Shon, 2000; Schmidt et al., 2000; LeBreton et al., 2014; Sackett, 2014; Viswesvaran et al., 2014; Salgado et al., 2016). For example, the meta-analyses of Viswesvaran et al. (1996); Salgado et al. (2003), and Salgado and Tauriz (2014) found, with independent databases, that the average observed interrater reliability was 0.52, although the interrater reliability of job performance ratings in validity studies of personnel selection can be higher (around 0.64) in civilian occupations (Salgado and Moscoso, 1996). Nevertheless, even this higher interrater reliability is smaller than the reliability of frequently used objective performance measures, such as work samples, production records, and grades (Schmidt, 2002). For example, Hunter (1983b; see also Schmidt et al., 1986) found that the average reliability of work sample tests was 0.77 in eight occupations with a cumulative sample of 1,967 incumbents. With regard to production records, Hunter et al. (1990) found that the average reliability of output measures on non-piece-rate jobs was 0.55 for 1 week, 0.83 for 4 weeks, and 0.97 for 30 weeks. Judiesch and Schmidt (2000) found that the average reliability of output measures on piece-rate jobs was 0.80 for a week, 0.94 for 4 weeks, and it was practically perfect over 30 weeks. Salgado and Tauriz (2014) found an average reliability coefficient of 0.83 for production data, based on seven studies. Concerning to grades, Salgado and Tauriz (2014) found a reliability coefficient of 0.80 and, more recently, Beatty et al. (2015) found a reliability coefficient of 0.89. As a whole, this evidence shows that the supervisory performance ratings appear to be less reliable than the objective criteria. Therefore, independent meta-analyses using criteria type as a moderator variable seem to be advisable.

In addition, Hunter (1980, Hunter, 1983a, 1986; Hunter and Hunter, 1984) demonstrated the moderator role of job complexity on the validity of GMA for the combinations GMA-job proficiency and GMA-training, but it remains unclear if job complexity shows a similar moderating effect for the combinations of GMA and specific criteria. In other words, we do not know if the role of job complexity as a moderator variable of the GMA validity generalizes to the specific criteria used in the GATB validity studies.

In summary, there seems to be a need for a new meta-analysis that examines the validity of GMA as a predictor of several criterion measures not included (or not included as separate categories) in previous meta-analyses: production records, work samples, job performance ratings, instructor ratings, and GPA. The present meta-analysis is, therefore, the first one that examines the validity of the GMA for these criteria comparatively. As the majority of GATB validity studies have used supervisory ratings of performance as a criterion, this criterion will serve as the reference framework for the comparison of the validities against the other criteria. Consequently, the three following research questions can be posed:

Research Question 1: Is the validity of the GMA composites the same for the various measures of job performance and training success? In other words, do GMA composites predict job performance ratings, production records, and work sample tests equally well? Do GMA composites predict grades and, instructor ratings equally well?

Research Question 2: What is the interrater reliability of the overall job performance ratings used in GATB validity studies? Is it similar or lower than the reliability of the other criteria?

Research Question 3: Does the role of job complexity as a moderator of the validity of GMA generalize to the combination of GMA and specific criteria?

## Comparison of the Validity of the GMA Composites

As in Table 1, the GATB includes several measures for assessing eight specific cognitive abilities and General Learning Ability (G; a.k.a. General Mental Ability or General Intelligence), which is a cognitive ability composite. Clustering some of the specific abilities, Hunter (1983a) created a new GMA composite named GVN. The sum of the aptitudes G, V, and N produces the GVN composite. However, an alternative GMA composite (i.e., G) was already included in the GATB, but Hunter (1983a) did not examine the validity of the G composite. At present, there is no meta-analytic evidence of the validity of G composite.

Concerning G and GVN cognitive ability composites of the GATB, there are two relevant differences among them. The first one is that the GVN composite includes test 2 (computation), but the G composite does not contain it. The other difference is that the vocabulary test and the arithmetic reasoning test are scored twice in the GVN composite but only once in the G composite. However, there is no technical reason for this duplication as G is not defined (and measured) independently of the V, N, and S abilities. Beyond the differences mentioned, there are no others between G and GVN composites. However, it is not known whether the GVN composite shows equal or better operational validity than the G composite as an estimate of GMA.

Schmidt (2002, 2012) posited that a composite of two, three or more specific aptitudes (e.g., verbal, numerical, and spatial) is a *de facto* measure of GMA and that, after one controls for GMA, the specific aptitudes make no incremental contribution to the prediction of job performance and training success over and above the contribution of GMA. Based on this, the G composite as measured by the GATB is a measure of GMA, and the only difference from Hunter's GVN composite is that this last one includes an additional test (computation test), but not a different aptitude. Therefore, the potential difference in criterion validity between G and GVN would be due to the computation test and also the method that Hunter used to sum the abilities. In other words, if one controls for G, the GVN composite would not show incremental validity for predicting job proficiency and training success criteria over and above G.

Both Hunter and Hunter (1984) and the NAS panel (Hartigan and Wigdor, 1989) focused on the validity of GVN, but neither Hunter and Hunter nor the NAS panel estimated the validity of G composite separately. Therefore, it remains unexamined whether GVN shows more, equal, or less validity than G. Any potential differences between GVN and G validities would indirectly indicate which composite serves as a better estimate of GMA. This question is relevant because if there is no difference in validity between the GVN and G composites, this last one should be the preferred composite. If there are differences, they will show which composite should be the preferred option.

In summary, using the GATB tests, it is possible to create at least two GMA composites, i.e., G and GVN. Because the G composite already includes V, N, and S, the examination of the validity of G alone seems relevant to knowing the gains in the validity (incremental validity) achieved with the addition of the computation test in the GVN composite of GMA. This line of reasoning allows us to pose the second research question:

Research Question 4: What is the validity of G and GVN composites for predicting job proficiency and training success criteria?

## Range Restriction Issues

A third relevant issue has to do with range restriction (RR) and its possible correction. For an assessment procedure, there is RR when the standard deviation (SD) of the sample (i.e., of the restricted group) is smaller than the standard deviation (SD) of the population (i.e., of the unrestricted group). The RR can be direct or indirect. The first one happens when the individuals have been selected directly on the test scores. The indirect RR happens when the selection is made on a third variable that is correlated with the assessment procedure. Both forms of RR, direct and indirect, affect the validity coefficients in two senses. First, RR causes an underestimation of the validity, and the more severe the RR is, the greater the underestimation will be. Second, RR produces artifactual variability in the validity coefficients.

All the formulas to correct for RR require knowing the ratio between the SD of the restricted group (i.e., incumbents) and the SD of the unrestricted group (i.e., applicants). This last parameter is unknown in all validity studies included in the GATB database, but it can be estimated. Using a basic formula from analysis of variance to obtain the total variance of the population, Hunter (1983a; see also Feldt and Qualls, 1998; Schmidt et al., 2008) estimated that the unrestricted SD was 61.37 for the composite of GVN. Using this value, Hunter (1983a) estimated the RR ratio (homogeneity coefficient *u*) of the individual studies and found that the average *u-*values were 0.67 and 0.60 for the job proficiency studies and training success studies, respectively.

The NAS panel (Hartigan and Wigdor, 1989) did not correct for range restriction because they did not have the unrestricted SD and because they disagreed with Hunter's assumption that the applicant pool was the entire workforce. Moreover, according to the NAS panel, some findings suggested that the applicant pool for specific jobs could be more restricted than the applicant pool for the entire workforce. Consequently, Hunter's method might overcorrect, according to the NAS panel view. This issue was empirically examined by Sackett and Ostgaard (1994), who showed that the SDs of job-specific pools and the SD of national norms did not differ significantly in the case of a cognitive ability test (3% smaller than national norms for the least complex jobs and 10% on average for the rest of jobs). This finding strongly supported Hunter's method and his assumption for estimating the unrestricted SD. In connection with this issue, a study of Hoffman (1995) is relevant too. He found that the samples of experienced applicants and inexperienced applicants showed similar SDs. Besides, Hoffman concluded that the *u*-estimates based on the national norms were only 0.9% larger than the *u*-estimates based on experienced applicants and that they were identical to the estimates based on the inexperienced applicants, which totally supported Hunter's (1983a) assumption of the comparability of the applicant and national norms.

Recently, Berry et al. (2014) posited a new challenge to Hunter (1983a) *u*-coefficients. Berry et al. (2014, p. 27, Table 3) estimated the range restriction of the GMA composite of the GATB for three racial/ethnic groups, and they found *u*-values of 0.89, 0.86, and 0.85 for Whites, African-Americans, and Hispanics, respectively. The sample-size-weighted average *u* was 0.88 (*N* = 36,926).These values show considerably less range restriction than the 0.67 *u*-value found by Hunter (1983a) and, therefore, their effects on the validity of GMA are smaller. If these *u* estimates represent the proper RR values for the GATB database, then Hunter (1983a) and Hunter and Hunter (1984) might have overestimated the GMA validity. Thus, it seems convenient to re-estimate the RR distributions of the GATB, because the estimation of the GMA range restriction is an extremely relevant issue to establish its operational validity. This fact inspires the next research question.

Research Question 5: What is the extent of range restriction (u-value) for GMA composites in the GATB studies across job complexity levels and the five criteria of job performance and training success?

**Table 3:** Population descriptive statistics of GATB cognitive abilities and GMA composites.

**Ability**	**Mean*_***app***_***	**SD*_***app***_***	**Mean*_***inc***_***	**SD*_***inc***_***
G	100.06	19.48	100.00	18.30
V	98.54	16.90	99.20	17.30
N	97.93	18.46	97.50	19.10
S	101.84	20.53	100.10	20.00
GVN	296.52	55.25	–	–

*The descriptive statistics for applicants were estimated using the Hunter (1983a) method. The descriptive statistics for the incumbents were taken from the Manual for the USES General Aptitude Test Battery, section III: Development, Tables 6–9, p. 36*.

## Aims of this Study

In summary, the Manual of the GATB (U.S. Department of Labor, 1970), the aricle of Bemis (1968), and a cursory examination of the technical reports of the GATB show three points. First, a variety of criterion measures were used, including supervisory ratings of job performance, production records, work sample tests, instructor ratings, grade point average (GPA), and training grades. Second, some technical reports included information about the interrater reliability of job performance ratings, and, consequently, an empirical distribution of the reliability for this criterion might be created, avoiding the necessity to assume the reliability, as was done for instance by Hunter and Hunter (1984) and Schmidt et al. (2008). Third, some technical reports included information about the reliability of production records, grades, and instructor ratings, which permits to create empirical distributions of the reliability of these criteria. Fourth, the technical reports included the mean, standard deviation, and the observed validity of each aptitude included in the GATB. This information allows (a) to calculate the validity of the GVN composite and to compare it with the validity of the G composite, and (b) the means and standard deviations permit to empirically develop range restriction distributions (mean and SD of *u*-values) for the combinations GMA-specific criterion.

Consequently, this meta-analytic effort has had five goals. The first goal has been to examine whether the validity of the GMA composites of the GATB is similar or not for the specific criteria mentioned above. The second objective has been to determine whether the moderating effect of job complexity on GMA validity can be generalized to some unexamined criteria (e.g., work sample tests, production records, grades, and instructor ratings). The third goal has been to establish the reliability of the various criteria used in the GATB validity studies. The fourth goal has been to know if the two GMA composites of the GATB, i.e., G and GVN, show similar validity magnitudes. Finally, the fifth goal has been to develop the empirical distribution of range restriction for each predictor-criterion combination.

## Method

### Database and Procedure

The target population was the validation studies conducted by the U.S. Employment Service to estimate the criterion-oriented validity of the GATB. These studies were carried out over the period 1950–1985. An important characteristic of these studies is that there is a written technical report for each study. These technical reports are currently available to researchers in the ERIC database (www.eric.ed.gov). Typically, each technical report contains between 12 and 20 pages and includes information on the occupation name, D.O.T. code, job description, sample, means and SDs of the GATB abilities, type of criteria, validation design, criterion reliability, and validity coefficients for the nine abilities measured by the GATB. Therefore, the dataset for this meta-analysis consisted of all the written technical reports of the GATB currently available in the ERIC database.

**Figure 1:**
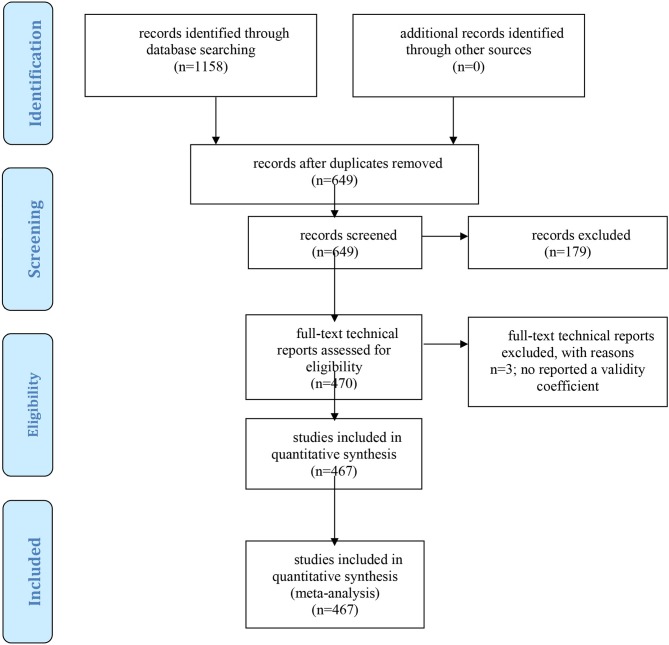
PRISMA flow diagram of excluded and included studies.

We conducted electronic searches in the ERIC database in three periods: August 26th, 2016 to September 26Th, 2016; February 27th 2017 to March 28th, 2017, and January 12th, 2018 to January 30th, 2018. In these searches, we used the following keywords: “technical report” and “report” in combination with “GATB,” “USES aptitude test battery,” “USTES aptitude test battery,” “USES specific aptitude test battery,” and “USES general aptitude test battery.” With this strategy, we obtained 1,158 references and we examined the content of each document. We excluded the references that did not report validity estimates and the duplicated references, and, finally, we have been able to locate and collect 467 technical reports that included 630 independent samples (i.e., validation studies). The list of the technical reports has been added as Supplementary Material. This meta-analysis conforms to the Meta-Analysis Reporting Standards (MARS) specified in the Publication Manual of the American Psychological Association (2010; available at https://apastyle.apa.org/manual/related/JARS-MARS.pdf), and the Preferred Reporting Items for Systematic Reviews and Meta-analysis (PRISMA) guidelines (Moher et al., 2009). The PRISMA flowchart is shown in Figure 1. In accordance with the MARS guidelines and the PRISMA guidelines, we have added as Supplementary Material seven files. One file contains the full list of references, and six files contain the following information from each study: (a) ERIC database code, (b) job complexity level, (c) sample size, (d) observed validity of g composite; (e) observed validity of GVN composite; (f) range restriction *u*-value of g composite; and (g) range restriction *u*-value of GVN composite.

For each study, we recorded the following information, if available: (1) sample characteristics, such as size, gender, ethnic group, age, education, and experience; (2) occupation and related information (e.g., name, DOT code); (3) GATB battery used; (4) criterion type; (5) criterion reliability; (6) mean and SD of the GATB abilities; (7) correlation between GATB abilities and the criterion (or criteria when more than one was used); and (8) number of raters when more than one was used. When a study contained conceptual replications (i.e., two or more rating criteria), linear composites with unit weights for the components were formed. Linear composites provide estimates that are more construct-valid than the use of the average correlation. Nunnally (1978) and Schmidt and Hunter (2015) provided Mosier's formula for the correlation of variables with composites.

We estimated the reliability of the codification with the correlation between two coders with experience conducting meta-analyses. The reliability between coder A and coder B was 1 for criterion (performance vs. training), for criterion type (work sample test, production records, job performance ratings, instructor ratings, GPA, and work sample test), design type (concurrent vs. predictive), and sample size, and 0.90 for job complexity. Consensus solved divergences after going back to the job description of the technical report and the D.O.T. descriptions for the specific jobs.

### Job Complexity

The next step was to classify all the jobs included in the studies according to their job complexity level. Job complexity is probably the most critical moderator of the validity of GMA tests (Hunter, 1983a, 1986; Hunter and Hunter, 1984; Salgado et al., 2003; Schmidt et al., 2008). Recent evidence shows that job complexity also moderates the validity of forced-choice personality inventories (Salgado, 2017b). We used three levels of job complexity, following the classification scheme of Hunter (1986; see also Hunter et al., 1990; Schmidt et al., 2008). Occupations classified as being of high complexity consisted of the jobs coded 0 and 1 on the Data dimension and jobs with code 0 on the Things dimension. The medium level of job complexity consisted of jobs with codes 2, 3, and 4 on the Data dimension. The low level of job complexity consisted of jobs with codes 5 and 6 on the Data dimension and jobs with a code of 6 on the Things dimension. This three-level job complexity classification is the same as that used by Hunter (1986), Salgado et al. (2003), and Schmidt et al. (2006, 2008).

Finally, after we examined the technical reports individually, and we recorded their characteristics, the next step was to apply the meta-analysis method of Schmidt and Hunter (2015). This method estimates the operational and true validities of the predictor, and how much of the observed variance of validity across studies is due to artifactual errors. The artifacts considered in the current meta-analysis were sampling error, criterion reliability, predictor reliability, and RR in predictor scores. Because studies rarely provide all the information required to individually correct the observed validity for the last three artifacts, the most common strategy is to develop specific distributions for each of them (Salgado et al., 2003; Schmidt and Hunter, 2015). Some of these artifacts reduce the correlations below their operational value (e.g., criterion reliability and range restriction), and all of them produce artifactual variability in the observed validity (Salgado et al., 2003; Schmidt and Hunter, 2015). In this meta-analysis, we corrected the observed mean validity for criterion reliability and RR in the predictor in order to obtain the operational validity (which is of interest for personnel selection and academic decisions), and we corrected the operational validity for predictor reliability to obtain the true score correlation (which is of interest for modeling the theoretical relationship between predictor and criteria).

### Artifact Distributions

#### Range Restriction Distributions

To obtain the degree of range restriction in each study, we followed Hunter's (1980, Hunter, 1983a) method. Nevertheless, the steps of the method are not the same for the specific abilities as for the GMA (i.e., GVN) composite. First, we will explain the process for the specific abilities and then the process for the GMA composite.

The technical reports contained the SD of G, V, N, and S tests for each validity study. This SD is the restricted group SD for these abilities. Next, to obtain the SD of the working population, we took the following steps. First, we calculated the variance of the means and the mean of the variances for each ability. The sum of these two values gives the variance of the population. The square root of the variance gives the SD of the unrestricted group or the SD of abilities for the whole working population. Dividing the SD of the restricted group by the SD of the population, we obtain the *u*-value for each ability in every single study.

In the case of the GVN composite, we followed the same method, but it needed some additions. First, to obtain the GVN score of each study, we summed the mean of G, V, and N in each study. These values make it possible to the variance of the GVN means. Second, to obtain the GVN variance of every single study we used the formula for the variance of a composite of three unweighted measures (see, for instance, Guilford and Fruchter, 1978, pp. 351–352):

$$\begin{array}{l} S_{gvn}^{2}=s_{g}^{2}+s_{v}^{2}+s_{n}^{2}+2r_{gv}s_{g}s_{v}+2r_{gn}s_{g}s_{n}+2r_{vn}s_{v}s_{n} \end{array}$$

This formula requires three covariances. To calculate these covariances, we used the observed intercorrelations between G, V, and N given in the manual of the GATB (U.S. Department of Labor, 1970; section III, p. 34). These correlations were 0.81 for G-V, 0.86 for G-N, and 0.67 for V-N. Third, we calculated the square root of the variance of each study to obtain the SD of the restricted group. Next, we calculated the mean of the GVN variances. The square root of the sum of the variance of the means plus the mean of the variances gives the population SD of the GVN composite. Now, dividing the SD of each study by the SD of the population (SDp), we obtain the *u-*value for GVN in every single study.

Table 3 reports the population's SD values for the GMA composites. For example, Hunter (1983a) indicated that the SDp of GVN was 61.63 in the case of job proficiency studies, and we found an SDp of 55.25, which is about 10% smaller. This difference is of no great practical importance, and it is probably because the number of studies (and the total sample size) is not the same in Hunter (1983a) and the present study. In Table 4 appear the *u*-values of range restriction for the GMA composites.

**Table 4:** Range restriction distributions of GMA composites across job complexity levels.

	**High**		**Medium**		**Low**	
**Ability**	**ū**	**SD*u***	**ū**	**SD*u***	**ū**	**SD*u***
**JOB PERFORMANCE RATINGS**						
G	0.68	0.09	0.75	0.11	0.75	0.11
GVN	0.71	0.09	0.77	0.10	0.78	0.10
	*k* = 41		*k* = 214		*k* = 172	
**INSTRUCTOR RATINGS**						
G	0.68	0.01	0.74	0.09	0.77	0.10
GVN	0.69	0	0.75	0.08	0.76	0.09
	*k = 2*		*k = 33*		*k = 8*	
**GRADES**						
G	0.6	0.07	0.64	0.08		
GVN	0.63	0.07	0.66	0.07		
	*k* = 36		*k* = 31			
**PRODUCTION RECORDS**						
G			0.68	0.11	0.73	0.10
GVN			0.73	0.09	0.77	0.07
			*k* = 7		*k* = 18	
**WORK SAMPLE TEST**						
G			0.65	0.13	0.70	0.08
GVN			0.68	0.11	0.74	0.07
			*k = 9*		*k = 2*	

*K, number of coefficients*.

#### Predictor Reliability

For the GMA composites, the appropriate coefficients are test-retest estimates with parallel forms (Schmidt and Hunter, 1999; Schmidt et al., 2003). However, in the absence of these coefficients, test-retest coefficients (with the same test form given twice) and internal consistency coefficients (e.g., Cronbach's alpha) are an acceptable alternative. The population reliability of the G composite and the specific cognitive abilities was taken from the coefficients reported in the Manual of the USES General Aptitude Test Battery. The population reliability of GVN composite was estimated using the Spearman-Brown formula for obtaining the reliability of a composite. The value was 0.94 for GVN.

As the reliability of the GMA measures is lower in the samples used in the validity studies than in the population due to range restriction, a coefficient of the restricted reliability of each ability and the GMA composites was estimated for each independent study using the formula $r_{xx}=1-U^{2}\left(1-R_{xx}\right)$ of Madnusson's (1967, p. 75), where *U*^2^ is *1/u*^2^ and *R*_*xx*_ is the unrestricted reliability. This formula gives the same value as the formula 3.17c of Schmidt and Hunter (2015, p. 127). This formula permits the creation of specific predictor reliability distributions across job complexity levels and criterion type combinations. Table 5 reports the predictor reliability distributions.

**Table 5:** Reliability distributions of GMA composites across job complexity levels.

	**High**		**Medium**		**Low**	
**Ability**	**${\bar{r}}_{xx}$**	**SD*rxx***	**${\bar{r}}_{xx}$**	**SD*rxx***	**${\bar{r}}_{xx}$**	**SD*rxx***
**JOB PERFORMANCE STUDIES**						
G	0.82	0.05	0.85	0.05	0.85	0.05
GVN	0.88	0.03	0.89	0.03	0.90	0.03
	*k* = 41		*k* = 213		*k* = 173	
**INSTRUCTOR RATINGS STUDIES**						
G	0.83	0.01	0.84	0.05	0.86	0.03
GVN	0.87	0	0.89	0.03	0.89	0.02
	*k* = 2		*k* = 33		*k* = 8	
**GRADES STUDIES**						
G	0.77	0.06	0.8	0.06		
GVN	0.84	0.03	0.86	0.03		
	*k* = 36		*k* = 31			
**PRODUCTION RECORDS STUDIES**						
G			0.82	0.05	0.84	0.03
GVN			0.88	0.03	0.90	0.02
			*k* = 7		*k* = 18	
**WORK SAMPLE TEST STUDIES**						
G			0.78	0.09	0.83	0.04
GVN			0.86	0.05	0.90	0.02
			*k* = 9		*k* = 2	

*K, number of coefficients*.

#### Criterion Reliability Distributions

In the case of several studies, two validity coefficients were computed on the same sample using two or three measures of overall job performance (e.g., two supervisors rated the incumbents and correlations were calculated for each supervisor). Therefore, they were not statistically independent. According to Schmidt and Hunter (2015), the most appropriate procedure in such cases is to compute the correlation of the predictor with the *sum* of the performance measures. However, calculating this correlation for studies that did not report it requires knowledge of the correlations between the performance measures. When this information was not reported, the average correlation within the sample (along with the average sample size) was estimated. This procedure causes a downward bias in validity estimates (Schmidt and Hunter, 2015). In a small number of cases, the technical report pointed out that one measure was more construct valid than the others. In these cases, the validity of that criterion measure was used in the meta-analysis, and an average correlation was not computed.

In this meta-analysis, we used five types of criteria: (1) supervisory ratings of job performance, (2) production data, (3) work-sample tests, (4) grades (e.g., marks, grade point average), and (5) instructor ratings. We made this choice for two reasons: (1) previous meta-analyses of GATB used some of these types of criteria (e.g., job performance and grades), and one of the objectives of this research was to provide a comparison with those meta-analyses; consequently, it was essential to retain the same criteria; (2) other criteria, such production records, work-sample tests, supervisory ratings, GPA, and instructor ratings were not used in previous meta-analyses, but they were present in the current database; therefore, it was possible to carry out meta-analyses in these cases, and they would be a contribution to the literature. Some studies did not contain information about the criteria reliability. Therefore, we developed empirical distributions for the five criteria. Fortunately, a significant number of studies provided reliability coefficients for estimating criterion reliability. As a whole, we found 68 independent reliability coefficients. Table 6 presents the distributions used in this meta-analysis.

**Table 6:** Criterion reliability distributions.

**Criterion**	***N***	***K***	***r_***yy***_***	**SD_**y**_**
Supervisor ratings^a^	2,807	39	0.70	0.124
Instructor ratings^a^	444	6	0.71	0.076
Production records^b^	211	7	0.78	0.138
Work sample tests^b^	226	16	0.84	0.168
GPA—Grades^c^	780	12	0.82	0.074

a *Interrater coefficient*.

b *Test-retest reliability*.

c *Internal consistency coefficient*.

For job performance ratings, the coefficient of interest when a meta-analysis of random effects is carried out is interrater reliability (Hunter, 1986; Schmidt and Hunter, 1996; Sackett, 2003). This is because if this type of reliability is used in the correction for attenuation, it will correct most of the unsystematic errors in supervisor ratings (Hunter and Hirsh, 1987), although not all researchers agree with this point of view (e.g., Murphy and De Shon, 2000). We found 39 independent studies reporting interrater coefficients of supervisory performance ratings. All the coefficients were collected for research purposes, and the USES analysts assisted the raters (e.g., supervisors, foremen, directors) during the process of rating the employees. This characteristic is very noteworthy as it was not frequently mentioned in the research literature that all the criterion ratings were collected for research purposes. The average observed interrater coefficient was 0.70 (*SD* = 0.12). This average coefficient is considerably larger than the coefficient found in the meta-analyses of Viswesvaran et al. (1996) and Salgado et al. (2003), Salgado and Tauriz (2014). Also, it is larger than the value assumed by Hunter in his meta-analyses (Hunter, 1980, 1983a, 1986; Hunter and Hunter, 1984).

The average reliability of the objective productivity measures was 0.78 (*SD* = 0.14), based on seven studies. This value is similar to the one found by Hunter et al. (1990), Judiesch and Schmidt (2000), and Salgado and Tauriz (2014).

In the case of training proficiency criteria, six studies reported the interrater reliability of instructor ratings. The average reliability was 0.71 (*SD* = 0.076). We have also found 12 coefficients of GPA reliability, which produced an average reliability coefficient of 0.82 (*SD* = 0.074). This last value is similar to the one reported by Salgado and Tauriz (2014), but slightly lower than the value found by Beatty et al. (2015).

No studies reported the reliability of work sample tests but, based on cumulative research findings (Hunter, 1983b; Hunter et al., 1990; Judiesch and Schmidt, 2000; Roth et al., 2005), we estimated the internal consistency, the interrater reliability, and the test-retest reliability of the work sample tests. The average internal consistency was 0.81 (*K* = 18), the test-retest reliability was 0.84 (*K* = 5; *SD* = 1.6), and the interrater reliability was 0.84 (*K* = 16). As the interrater reliability and the test-retest reliability are the most appropriate coefficients for the work-sample tests, and they were 0.84, this value was used for these criterion measures.

### Meta-Analysis Method

We used the psychometric meta-analysis methods developed by Schmidt and Hunter (2015) and a software program developed by Schmidt and Le (2014) to implement them. This software includes some recent advances to correct for indirect range restriction (IRR). The software program includes both the new refinements and older advances and refinements, such as the use of mean *r* instead of the study observed *r* in the formula for sampling error variance and a new non-linear range-correction procedure (Schmidt et al., 1993; Law et al., 1994a,b). We were interested in the relationship between the GMA composites and the criteria, both as theoretical constructs and as operational predictors. Therefore, we report both the operational validity and the true correlation. In summary, we correct the observed validity for criterion reliability and IRR to obtain the operational validity, and we will correct for predictor unreliability to obtain the true correlation. The observed variance was corrected for by four artifactual errors: sampling error, criterion, and predictor reliability, and IRR.

## Results

The results of the meta-analyses for the GMA composites-criteria-job complexity levels appear in Tables 7–**11**. In these Tables, from left to right, the first two columns show the total sample size (*N*) and the number of independent validation studies (*K*). The next four columns represent the observed validity weighted by study sample size (*r*), the standard deviation of the observed validity (SD_*r*_), the sampling error variance (SEV), and the percentage of observed variance accounted (%EV) for by the four sources of artifactual errors (i.e., sampling error, criterion reliability, range restriction in predictor, and predictor reliability). The next four columns show the operational validity (i.e., *r*_*o*_; observed validity corrected for indirect range restriction and criterion reliability), the standard deviation of the operational validity (SD*r*_*o*_), the score correlation validity (i.e., ρ, the operational validity corrected for predictor reliability), and the standard deviation of the true score correlation. Finally, the last two columns represent the 90% credibility value of the operational validity and the 95% confidence interval of the score correlation. In these meta-analyses, we report both the operational validity and the true score correlation because they serve different goals. The operational validity is the coefficient to be used for predicting the various criteria in applied settings (e.g., for hiring employees or recruiting students). True score correlation represents the theoretical correlation between the predictor and the criterion in the absence of artifactual errors (it can also be called the construct level correlation). Consequently, the true score correlation is used for modeling the theoretical relationships between predictors and criteria (Salgado et al., 2003). Although both estimates are of interest, we will concentrate on the true score correlation in the following comments.

**Table 7:** Validity of general mental ability composites for predicting work sample tests.

**Ability**	***N***	***K***	***r***	**SD*_***r***_***	**SEV**	**%VE**	***r_***o***_***	**SD*_***o***_***	**ρ**	**SD*_**ρ**_***	**90CV*r_***o***_***	**95CIρ**
**MEDIUM COMPLEXITY**												
G	562	9	0.31	0.122	0.014	100	0.55	0.000	0.57	0.000	0.55	0.43/0.72
GVN	556	8	0.32	0.142	0.012	71	0.52	0.101	0.53	0.104	0.39	0.37/0.70
**LOW COMPLEXITY**												
G	137	2	0.25	0.141	0.013	70	0.40	0.111	0.41	0.117	0.25	0.08/0.75
GVN	137	2	0.28	0.171	0.013	47	0.41	0.165	0.42	0.170	0.20	0.07/0.78

*N, total sample size; K, number of validities; r, weighted-sample observed validity; SDr, weighted-sample observed standard deviation; SEV, sampling error variance; %VE, percentage of variance accounted for by all artifacts; r_o_, operational validity; SD_o_, standard deviation of the operational validity; ρ, true score correlation; SD_ρ_, standard deviation of ρ; 90CV, credibility value of 90% of r_o_; 95CI, confidence interval of 95% of ρ*.

Table 7 shows the results for the combinations of GMA composites, work sample test, and job complexity levels. In this case, we found studies for the medium and low complexity levels only. For the medium complexity level, the true score correlation estimates were 0.57 for G and 0.53 for GVN. Therefore, the best GMA composite was G for predicting work sample tests in this level of job complexity. The 90% credibility values were positive and substantially different from zero (0.39 and 0.55 for GVN and G, respectively), which demonstrated robust evidence of validity generalization. The percentage of explained variance was 71% and 100%, respectively. For the low complexity level, the number of studies was two; therefore, the findings for this complexity level should be considered with caution. The true score correlations were 0.41 for G and 0.42 for GVN. In the two cases, the 90% CV was positive and substantially different from zero, which indicated validity generalization evidence for this complexity level. The variance accounted for by the artifactual errors was 70 and 47% for G and GVN, respectively. Therefore, taking the results for the two job complexity levels as a whole, the findings showed that GMA predicted work sample tests very efficiently and that job complexity moderated the magnitude of the validity.

Table 8 shows the results for the combinations of GMA composites, production records, and job complexity levels. As in the case of work sample tests, for the criterion of production records, we found studies for the medium and low complexity levels only. For the medium level of job complexity, the true score correlations of the GMA composites were 0.39 for G and 0.29 for GVN. Again, although with a different criterion, the best GMA composite was G. The 90% credibility values were positive and substantially different from zero, which again demonstrated evidence of validity generalization. All the observed variance was explained by the artifactual errors. For the low complexity level, the true score correlation was 0.21 for both G and GVN. In the two cases, the 90% CV was positive (0.06 and 0.07, respectively), which indicated validity generalization evidence for this complexity level. The variance accounted for by the artifactual errors was 82% for G and 78% for GVN.

**Table 8:** Validity of general mental ability composites for predicting production records.

**Ability**	***N***	***K***	***r***	**SD*_***r***_***	**SEV**	**%VE**	***r_***o***_***	**SD*_***o***_***	**ρ**	**SD*_**ρ**_***	**90CV*r_***o***_***	**95CIρ**
**MEDIUM COMPLEXITY**												
G	357	7	0.21	0.089	0.018	100	0.37	0.000	0.39	0.000	0.37	0.26/0.51
GVN	357	7	0.17	0.087	0.019	100	0.28	0.000	0.29	0.000	0.28	0.18/0.39
**LOW COMPLEXITY**												
G	1,222	24	0.12	0.156	0.020	82	0.20	0.105	0.21	0.110	0.06	0.10/0.32
GVN	1,222	24	0.14	0.159	0.019	78	0.22	0.110	0.21	0.113	0.07	0.11/0.31

*N, total sample size; K, number of validities; r, weighted-sample observed validity; SDr, weighted-sample observed standard deviation; SEV, sampling error variance; %VE, percentage of variance accounted for by all artifacts; r_o_, operational validity; SD_o_, standard deviation of the operational validity; ρ, true score correlation; SD_ρ_, standard deviation of ρ; 90CV, credibility value of 90% of r_o_; 95CI, confidence interval of 95% of ρ*.

Therefore, for the criterion of production records, the findings showed that GMA was an efficient predictor and that job complexity moderated the magnitude of the validity. It is important to note that, in comparison with the GMA validity for predicting work sample tests, the validity for predicting production records is considerably smaller. For instance, in the case of the G composite, the true score correlation is 50% larger for predicting work sample tests than for predicting production records in the medium level of job complexity, and it is practically double in the case of the low level of job complexity.

Table 9 shows the results for the combinations of GMA composites, job performance ratings, and job complexity levels. In this case, we found studies for the three levels of job complexity. For the high level, the true score correlations were 0.52 and 0.48 for G and GVN, respectively. Therefore, G was the best GMA composite for job performance ratings at the highest level of job complexity. The 90% credibility values were positive and substantially different from zero, which demonstrated evidence of validity generalization. The percentage of explained variance was 84% for G and 96% for GVN. For the medium level of job complexity, true validities were 0.46 and 0.43, for G and GVN, respectively. The 90% credibility values were positive and substantially different from zero, which showed evidence of validity generalization. The percentage of explained variance was 77% for both GMA composites. For the low complexity level, the true score correlation were 0.35 for G and 0.31 for GVN. The 90% CVs were positive and substantially different from zero, which indicated validity generalization evidence for this complexity level. The average explained variance for the artifactual errors was 85%. Therefore, taking the results for the three job complexity levels as a whole, the findings demonstrated that GMA predicted supervisory job performance ratings across the three levels of job complexity very efficiently. The validity evidence also showed that job complexity was a powerful moderator of true score correlation. On average, the validity for the medium complexity level was 29% greater than the validity for the low complexity level, and the validity for the high complexity level was 8% greater than the validity for the medium level. Another significant finding was that G, the simplest composite of GMA in the GATB, was consistently the best predictor of supervisory ratings across the three levels of job complexity.

**Table 9:** Validity of general mental ability composites for predicting supervisory performance ratings.

**Ability**	***N***	***K***	***r***	**SD*_***r***_***	**SEV**	**%VE**	***r_***o***_***	**SD*_***o***_***	**ρ**	**SD*_**ρ**_***	**90CV*r_***o***_***	**95CIρ**
**HIGH COMPLEXITY**												
G	3,003	45	0.28	0.132	0.013	84	0.50	0.078	0.52	0.082	0.40	0.45/0.59
GVN	3,003	45	0.28	0.122	0.013	96	0.47	0.038	0.48	0.039	0.42	0.42/0.54
**MEDIUM COMPLEXITY**												
G	17,977	214	0.27	0.125	0.010	77	0.44	0.086	0.46	0.09	0.33	0.43 /0.49
GVN	17,911	213	0.26	0.123	0.010	77	0.41	0.108	0.43	0.087	0.30	0.40/0.45
**LOW COMPLEXITY**												
G	11,855	178	0.20	0.135	0.014	81	0.34	0.089	0.35	0.094	0.22	0.31/0.38
GVN	11,855	178	0.19	0.127	0.014	92	0.30	0.122	0.31	0.055	0.28	0.28/0.34

*N, total sample size; K, number of validities; r, weighted-sample observed validity; SDr, weighted-sample observed standard deviation; SEV, sampling error variance; %VE, percentage of variance accounted for by all artifacts; r_o_, operational validity; SD_o_, standard deviation of the operational validity; ρ, true score correlation; SD_ρ_, standard deviation of ρ; 90CV, credibility value of 90% of r_o_; 95CI, confidence interval of 95% of ρ*.

Summarizing the results for the three job performance criteria, it can be concluded that: (1) GMA estimates predicted the three criteria efficiently; (2) GMA validity is larger for predicting work sample tests than for predicting supervisory performance ratings, and production records, and larger for predicting supervisor ratings than production records; (3) job complexity was a powerful moderator of the validity across the three levels, so that as job complexity increases validity increases; and (4) as a whole, the simplest GMA composite, G, was consistently the best predictor of the three criteria across the job complexity levels.

The results of the validity of the GMA composites for instructor ratings of training performance across the three levels of job complexity appear in Table 10.

**Table 10:** Validity of general mental ability composites for predicting instructor ratings.

**Ability**	***N***	***K***	***r***	**SD*_***r***_***	**SEV**	**%VE**	***r_***o***_***	**SD*_***o***_***	**ρ**	**SD*_**ρ**_***	**90CV*r_***o***_***	**95CIρ**
**HIGH COMPLEXITY**												
G	108	2	0.39	0.231	0.014	26	0.60	0.247	0.63	0.286	0.11	0.11/1.0
GVN	108	2	0.37	0.201	0.014	35	0.58	0.207	0.59	0.214	0.31	0.15/1.0
**MEDIUM COMPLEXITY**												
G	3,336	35	0.34	0.143	0.008	48	0.53	0.137	0.56	0.143	0.32	0.46/0.61
GVN	3,336	35	0.33	0.134	0.009	49	0.50	0.129	0.52	0.133	0.34	0.45/0.59
**LOW COMPLEXITY**												
G	605	8	0.36	0.110	0.010	97	0.55	0.025	0.58	0.026	0.52	0.46/0.70
GVN	553	7	0.32	0.088	0.010	100	0.49	0.000	0.51	0.000	0.49	0.41/0.61

*N, total sample size; K, number of validities; r, weighted-sample observed validity; SDr, weighted-sample observed standard deviation; SEV, sampling error variance; %VE, percentage of variance accounted for by all artifacts; r_o_, operational validity; SD_o_, standard deviation of the operational validity; ρ, true score correlation; SD_ρ_, standard deviation of ρ; 90CV, credibility value of 90% of r_o_; 95CI, confidence interval of 95% of ρ*.

For the high level, the true score correlation were 0.63 for G and 0.59 for GVN. The 90% credibility values were positive and different from zero, which showed evidence of validity generalization. The percentage of explained variance was 26% for G and 35% for GVN, which suggests that additional moderators can explain the observed variance. It is important to take into account that these results were obtained with two studies only, and, therefore, they should be considered provisional until additional studies can be added. For the medium level of job complexity, true validities were 0.56 for G and 0.52 for GVN. The 90% credibility values were positive and substantially different from zero, which showed evidence of validity generalization. The percentage of explained variance was 48% for G and 49% for GVN. Therefore, additional moderators can be expected. For the low complexity level, the true score correlation ranged from 0.58 for G and 0.51 for GVN. The 90% CVs were positive and substantially different from zero, which indicated evidence of validity generalization for this complexity level. The explained variance for the artifactual errors was 97% for G and 100% for GVN. Therefore, taking the results for the three job complexity levels as a whole, the findings demonstrated that GMA predicted instructor ratings of training performance across the three levels of job complexity very efficiently.

With regard to the moderating role of job complexity, the results were ambiguous for this criterion. On the one hand, the validity was larger for the high level of job complexity than for the medium and low levels. However, on the other hand, the average validity was practically identical for the medium and low levels of job complexity. Also, because the estimates for the high level of job complexity were based on only two coefficients, the best conclusion is that the findings are not conclusive about the moderation effect of job complexity for this criterion.

Table 11 shows the results for the combination of GMA composites, grades, and job complexity levels. In this case, we found studies for the high and medium job complexity levels only. For the high complexity level, the true score correlation of GMA composites were 0.65 and 0.57 for G and GVN, respectively. Therefore, the best GMA composite for predicting academic grades was G in this level of job complexity. The 90% credibility values were positive and substantially different from zero, which demonstrated robust evidence of validity generalization. The percentage of explained variance was 62% for G and 64%% for GVN. For the medium complexity level, the true score correlation were 0.67 for G and 0.59 for GVN. The 90% CV was positive and substantially different from zero in both cases, which indicated validity generalization evidence for this complexity level. The percentage of explained variance was 91 and 70% for G and GVN, respectively. Therefore, the results for the two job complexity levels showed that GMA predicted grades very efficiently and that job complexity moderated the magnitude of the validity. By a considerable margin, G was consistently better GMA predictor of grades than GVN.

**Table 11:** Validity of general mental ability composites for predicting grades.

**Ability**	***N***	***K***	***r***	**SD*_***r***_***	**SEV**	**%VE**	***r_***o***_***	**SD*_***o***_***	**ρ**	**SD*_**ρ**_***	**90CV*r_***o***_***	**95CIρ**
**HIGH COMPLEXITY**												
G	2,807	38	0.34	0.140	0.011	62	0.62	0.112	0.65	0.116	0.48	0.56/0.73
GVN	2,807	38	0.33	0.137	0.011	64	0.55	0.111	0.57	0.115	0.41	0.49/0.65
**MEDIUM COMPLEXITY**												
G	2,235	33	0.39	0.056	0.011	91	0.64	0.041	0.67	0.042	0.59	0.60/0.74
GVN	2,235	33	0.36	0.133	0.011	70	0.58	0.092	0.59	0.095	0.46	0.52/0.67

*N, total sample size; K, number of validities; r, weighted-sample observed validity; SDr, weighted-sample observed standard deviation; SEV, sampling error variance; %VE, percentage of variance accounted for by all artifacts; r_o_, operational validity; SD_o_, standard deviation of the operational validity; ρ, true score correlation; SD_ρ_, standard deviation of ρ; 90CV, credibility value of 90% of r_o_; 95CI, confidence interval of 95% of ρ*.

In summary, the results for the two training performance criteria indicate that: (1) GMA estimates predicted the two criteria very efficiently; (2) GMA validity is similar for both criteria; (3) job complexity moderates the validity of GMA composites slightly; and (4) as was found for job performance criteria, the simplest GMA composite, G, was consistently the best predictor for the two criteria across the job complexity levels.

As G was consistently the best predictor for the five criteria examined in this research, the last analysis in this section was to compute the validity of G for predicting job proficiency and training success. In this analysis, the validity for job proficiency was estimated as the combination of the validities for work sample tests, production records, and job performance ratings. The validity for training success was estimated as the combination of the validities for instructor ratings and grades. As reported in Table 12, the average validity across complexity levels was 0.44 for predicting job proficiency, and 0.39, 0.46, and 0.52 for low, medium, and high levels of job complexity, respectively. For training success, the average validity across complexity levels was 0.62, and it was 0.58, 0.61, and 0.64 for the low, medium, and high job complexity levels. In this case, job complexity was shown to be a relevant validity moderator of predictive validity, which was not so clear when instructor ratings and grades criteria were analyzed individually.

**Table 12:** Validity of general mental ability composites for predicting job proficiency and training success.

**Ability**	***N***	***K***	***r***	***r_***o***_***	**ρ**
**JOB PROFICIENCY**					
G—High	3,003	45	0.28	0.50	0.52
G—Medium	18,896	230	0.21	0.44	0.46
G—Low	2,807	204	0.19	0.32	0.39
G—All	35,113	479	0.21	0.40	0.44
**TRAINING SUCCESS**					
G—High	2,915	40	0.35	0.62	0.64
G—Medium	5,571	68	0.36	0.58	0.61
G—Low	605	8	0.36	0.55	0.58
G—All	9,091	116	0.36	0.59	0.62

*N, total sample size; K, number of validities; r, weighted-sample observed validity; r_o_, operational validity; ρ, true score correlation*.

## Discussion

### Main Findings

This research was a meta-analytic examination of the GMA validity carried out on the GATB for about 40 years. The meta-analyses were performed by examining the information contained in the written technical reports of the validation studies conducted by the USES. The examination of the whole set of written technical reports is a critical difference of the current study with respect to previous meta-analyses that used the GATB validity coefficients (e.g., Hartigan and Wigdor, 1989; Levine et al., 1996; Schmidt et al., 2008; Berry et al., 2014). Some of these meta-analyses used published articles, other meta-analyses used data recorded on a tape, and other meta-analyses used previous meta-analytic estimates, but they did not use the data contained in the technical reports. In comparison with Hunter's meta-analyses (Hunter, 1980, 1983a, 1986; Hunter and Hunter, 1984), the present research used a larger set of technical reports as we included the technical reports written after Hunter and Hunter's (1984) meta-analysis was conducted. This fact explains why the current research included 100 additional independent samples and that the total sample size is about 6,000 individuals larger than Hunter's meta-analyses.

The main goal of the meta-analysis was to provide an estimate of the validity of GMA as a predictor of five different performance and training criteria, including supervisory rating of job performance, instructor ratings, grades, production records, and work sample test criteria. The second main objective was to examine the moderating effects of job complexity on the GMA validity across these five criteria. The previous meta-analyses that examined the validity of GMA (e.g., Hunter, 1983a; Hunter and Hunter, 1984; Schmitt et al., 1984; Levine et al., 1996; Schmidt et al., 2008) unequivocally demonstrated that GMA was the best single-construct predictor of job proficiency and training success. However, these meta-analyses did not investigate the validity for some of the specific criteria used in the current study (e.g., production records, work sample tests, and instructor ratings). Therefore, these issues had remained meta-analytically unexplored until now. Furthermore, the validity of the two GMA composites provided by the GATB was not previously examined. Also, some previous meta-analyses used assumed artifact distributions (e.g., for predictor and criterion reliability, and range restriction) which can produce less accurate estimates of the GMA validity (e.g., Hunter, 1983a; Hartigan and Wigdor, 1989; Levine et al., 1996; Schmidt et al., 2008). Thus, two additional goals of the meta-analyses have been to examine and develop empirical distributions of the reliability of the criteria used in the GATB validity studies, and to develop empirical distributions of the GMA range restriction.

Probably the most significant contribution of this meta-analysis has been to provide an estimate of the validity of GMA for five performance and training criteria. With regard to this contribution, the results indicated, firstly, that GMA predicted all the criteria and showed validity generalization. Secondly, the results unequivocally demonstrated that the predictive efficiency of GMA is not the same for the five criteria. In the case of job performance criteria, GMA predicted work sample tests better than supervisory ratings and production records, and it predicted supervisor ratings better than production records. In the case of training performance, the two criteria, instructor ratings and grades, were predicted similarly well and showed the largest estimates of GMA validity across the five criteria. Therefore, the criterion type is a relevant variable in connection with the validity of GMA. Future validation studies should clearly specify what kind of criteria was used, as the validity estimates for one criterion type (e.g., supervisor ratings) should not be used as a subrogated estimate for other criterion types (e.g., work sample tests or production records). In third place, the most representative estimates of the GMA validity are 0.55, 0.37, 0.44, 0.53, and 0.64 for work sample tests, production records, supervisory ratings of overall job performance, instructor ratings, and grades, respectively. These are the validity estimates for the medium complexity jobs, as this job complexity level includes 62% of all the jobs in the U.S. economy (Schmidt and Hunter, 1998).

The second relevant contribution was to examine the GMA validity for the combination of criteria and job complexity levels. In the case of job performance criteria, the results demonstrated that job complexity was a relevant moderator of GMA validity for the three criteria. The findings were not as evident in the case of grades and instructor ratings. For instance, the validity is larger for the high level of complexity than for the other two levels in the case of instructor ratings, but the validity is smaller in the high level than in the medium level of complexity in the case of grades. To some extent, the ambiguity may be due to the small number of studies for some criterion-job complexity combinations. When the validity studies were aggregated into job proficiency studies and training success studies, the results replicated previous findings that showed that job complexity moderated the validity of GMA for predicting these two criteria (e.g., Hunter and Hunter, 1984; Salgado et al., 2003; Schmidt et al., 2008). According to our findings, the best estimates of the GMA operational validity for predicting job proficiency are 0.50, 0.44, and 0.32 for high, medium, and low complexity jobs, respectively, and the best estimates of GMA operational validity for training success are 0.62, 0.58, and 0.55, for high, medium and low complexity jobs, respectively. The estimates for predicting job proficiency are remarkably smaller than the estimates found by Hunter and Hunter (1984) and Hunter et al. (2006), which suggests that the use of these last estimates in personnel selection processes might overestimate job proficiency by a substantial degree.

A third contribution has been the comparison of the predictive validity for the two GMA composites derived from the GATB tests. In connection with this point, the significant finding has been that the simplest GMA composite (i.e., G) showed systematically larger validity than the alternative one. More specifically, the validity of G, the simplest composite, was larger than the validity of the GVN composite created by Hunter and Hunter (1984) in his meta-analysis. This finding suggests that the addition of tests does not necessarily produce an increase in the validity of a GMA composite. Therefore, from a cost-benefit perspective, G may produce greater economic utility than the alternative GMA composite that can be created from the GATB.

The fourth contribution has to see with the interrater reliability of supervisory ratings of overall performance. The interrater reliability found in the current research was 0.70, which is considerably larger than that of the previous studies; it is also remarkably greater than the reliability of 0.60 assumed by Hunter and Hunter (1984; see also Hunter, 1983a), and than the reliability assumed by Schmidt et al. (2008). The comprehensive meta-analysis of Viswesvaran et al. (1996) found that interrater reliability was 0.52 for overall job performance. This estimate was also obtained in the meta-analysis of the European studies of Salgado et al. (2003). A potential explanation for this divergence is that all the job performance measures included in the GATB validation studies were collected for research purposes, while this is not the case of the meta-analysis of Viswesvaran et al. (1996) and in Salgado et al. (2003) meta-analyses, which included also validation studies with job performance measures collected for administrative purposes.

The fifth contribution of this meta-analysis has been to develop empirically-derived distributions of range restriction for the combination predictor-criteria-job complexity levels. This contribution is relevant as it produces more accurate estimates of the GMA operational validity. Furthermore, we showed that (a) the restriction in range was not constant across the criteria, (b) range restriction (i.e., *u*-value) is slightly larger for G than for GVN, and (c) on average, Hunter's meta-analysis found *u*-values 15% smaller than the estimates for GVN of the current meta-analysis (0.67 vs. 77 for job proficiency studies and 0.60 vs. 69 for training success studies).

### Implications for the Research and Practice of Personnel Selection

The results of this series of meta-analyses have implications for the research and practice of personnel selection and the estimates of GMA validity, the range restriction values, and the interrater reliability of supervisory performance ratings.

Our GMA validity estimates were computed using empirically derived artifact distributions of reliability and range restriction -rather than on assumed distributions-, and the same studies that provided the validity coefficients provided the reliability coefficients and the range restriction values. Consequently, the GMA validity estimates found in the current meta-analysis are more accurate than the estimates found in the previous meta-analyses of the GATB validation studies (Hunter, 1983a; Hunter and Hunter, 1984; Schmidt et al., 2008). This fact suggests two practical implications. The first implication is GMA tests should be used in personnel selection processes for all types of jobs as GMA tests are excellent predictors of the occupational criteria examined, and they are probably the best single construct predictor. The second practical implication of our findings is that, as the validity estimates of GMA have smaller magnitude than was previously believed, other personnel selection procedures—used as supplements to GMA tests—can have more practical relevance, for instance, quasi-ipsative forced-choice personality inventories (Salgado and Tauriz, 2014; Salgado et al., 2015; Salgado, 2017b).

A third practical implication is that when an employer uses GMA tests in the selection processes, that employer should consider what criterion is to be predicted and to use the appropriate weight of GMA in a weighted combination with other selection procedures, as GMA tests do not predict all the occupational criteria equally.

Our findings also have research implications. A first research implication has to see with the comparison between our estimates of the validity of GMA and those of Hunter and Hunter (1984). In comparing the current findings with Hunter and Hunter's estimates of GMA validity, several points should be taken into account. First, Hunter and Hunter assumed 0.60 as the reliability of job proficiency, but as has been demonstrated, there were three different job performance criteria, and their reliability was considerably larger than 0.60. Second, over 50 validity coefficients were calculated with job performance ratings from two or more raters and, consequently, as the criterion was a more reliable one, the observed validity was higher. In the case of these fifty studies, there waas two potential analytic strategies in order to avoid biased corrected validity coefficients. The first strategy was to attenuate the observed validity using the interrater reliability for a single rater. The second strategy was to use the coefficient of higher interrater reliability when correcting the observed validity of these studies. For example, if two raters did the ratings, then an interrater reliability estimate of 0.82 should be used instead of 0.70; if three raters did the ratings, then an interrater reliability estimate of 0.87 should be used. However, Hunter (1983a) did not use these strategies and corrected all the observed validity using the interrater estimate of 0.60. Third, the data set contains 41 validity coefficients estimated using production records and work sample tests as a criterion, and these criteria are more reliable than job performance ratings. Fourth, the range restriction correction was a correction for indirect range restriction in the current study and a correction for direct range restriction in Hunter's meta-analysis. Fifth, Hunter used assumed reliability distributions for job proficiency and training success, and we derived the distributions empirically based on the information provided in the GATB studies. Sixth, Hunter used the same *u*-value for the three levels of complexity, but high complexity jobs showed more range restriction that medium and low complexity jobs. Therefore, as a whole, Hunter and Hunter (1984) could have underestimated the reliability criterion and overestimated the validity of GMA. Applying the reliability estimates found in the current study to Hunter and Hunter's study, the overall reliability of the job proficiency criterion would be around 0.73 (22% larger). Therefore, Hunter and Hunter (1984; see also Hunter, 1983a) overestimated the validity by about 10% for job proficiency.

The finding that the interrater reliability of supervisory ratings of overall performance was 0.70 has at least two research implications. Due to the fact that the interrater reliability can be larger if the performance ratings are gathered for research purposes, future meta-analyses that assume the interrater reliability of job performance ratings should consider the extent to which the criterion in the validation studies consisted of measures collected for research purposes and administrative purposes. Second, as some past meta-analyses of GMA validity that assumed unreservedly 0.52 as interrater reliability, they might have overestimated the validity of the specific selection procedure. As a whole, the importance of these findings deserves future research studies to clarify the real effect of the appraisal purpose of performance ratings (i.e., administrative vs. research) on the criterion reliability and predictor validity.

A final research implication of our findings has to do with the distributions of *u*-values found in this research. It is necessary to mention two specific aspects. First, the *u*-values found were similar to those found by Hunter (1983a), although they were 9% less restricted on average (0.67 vs. 0.73 for G) in the case of job proficiency; therefore, the effects of the RR on the criterion validity were, as a whole, slightly smaller in the present meta-analyses than in Hunter and Hunter (1984) meta-analysis. With regard to training, Hunter and Hunter (1984; see also Hunter, 1983a) *u*-estimate was 0.60 and our *u*-estimate are 0.67 (12% larger). Second, the current *u* estimates are remarkably smaller than the estimates reported by Berry et al. (2014) for the GATB. The divergence of the Berry et al. (2014) *u*-estimates with Hunter (1983a) and our estimates may be due to the use of different methodologies for obtaining the *u*-values, as Berry et al. (2014) took into account the variance between individuals but they did not consider the variance across occupations. Therefore, the methodology used to obtain the *u*-values must be clearly described in future studies.

As a suggestion for future research, we recommend that new studies should examine the relationship between GMA with less studied organizational criteria and behaviors such as innovative work performance (Harari et al., 2016) and job crafting (Ogbuanya and Chulwuedo, 2017).

### Limitations of the Present Meta-Analysis

This study also has several limitations. First, some cells contained a small number of cases. For example, work sample tests in low complexity jobs and instructor rating criterion in high complexity jobs contained four or fewer samples. Also, no studies were conducted for work sample tests and production records in high complexity jobs and for grades in low complexity jobs.

A second limitation is that, due to the content of the job performance measures (i.e., production records, work sample tests, and ratings), the validity studies of the GATB were all of them measures of task performance. Consequently, the validity estimates found here can serve as validity estimates of overall job performance and task performance only and not for other performance dimensions such as contextual (citizenship) performance or counterproductive work behaviors (CWB). The database used in this research does not permit conclusions about the generalization of the validity for these performance dimensions.

Some researchers and practitioners can see a limitation of this meta-analysis in the fact that many of the validity studies can be old and they may lack generalizability for the contemporary workplace. However, two findings run against this concern. First, The NAS Panel (Hartigan and Wigdor, 1989) found that the GMA validity does not decline over time, and Schmidt et al. (1988) concluded that the concerns that the GMA validity may decrease over time are unwarranted (see also Reeve and Bonaccio, 2011, for a review of the hypothesis of GMA degradation validity over time). The second finding is that the new version of the GATB (Forms E & F), renamed Ability Profiler (Forms 1 & 2), showed disattenuated correlations between old and new forms of the GATB ranging from 0.905 to 0.982 (Mellon et al., 1996). The disattenuated correlation between the olds forms and the new forms of the G composite was 0.955 and the correlation between the old forms and the new forms of the GVN composite was 0.968. Therefore, because the validity of GMA does not decline over time and because of the extremely high correlations between the abilities measures by the old and the new forms of the GATB, the operational validity estimates found for the old GATB forms serve as validity estimates for the new forms of the GATB.

## Conclusion

This meta-analytic re-examination of the validity studies conducted with the GATB showed that GMA is a consistent and valid predictor of five specific occupational criteria, including, supervisory ratings of overall job performance, production records, work samples tests, instructor ratings, and grades. In general, the validity magnitude of GMA was remarkably larger for the training criteria than for the performance criteria. Moreover, clustering the three performance criteria into a job proficiency criterion and clustering the two training criteria into a training success criterion, GMA showed to be a valid predictor of these two additional criteria, although the magnitude of the operational validity estimates for these two criteria is smaller than the previous estimates found by Hunter and Hunter (1984) and Schmidt et al. (2006) with the same database. The findings also revealed that job complexity moderated the GMA validity for predicting job performance criteria. An additional particularly relevant finding has been that the interrater reliability of the supervisory ratings of overall job performance was 0.70, which is remarkably larger than the interrater reliability found in previous meta-analyses.

In summary, we can conclude that the GMA is an excellent predictor of occupational performance criteria and that the best estimate of the operational validity of GMA is 0.50, 0.44, and 0.32 for high, medium, and low complexity jobs in the case of the job proficiency criterion and 0.62, 0.58, and 0.55 for the high, medium, and low complexity jobs in the case of the training success criterion.

## Data Availability Statement

All datasets generated for this study are included in the manuscript/Supplementary Files.

## Author Contributions

Both authors listed have made a substantial, direct and intellectual contribution to the work, and approved it for publication.

### Conflict of Interest

The authors declare that the research was conducted in the absence of any commercial or financial relationships that could be construed as a potential conflict of interest.
