# Supplementary material for: Meta-Analysis of the Validity of General Mental Ability for Five Performance Criteria: Hunter and Hunter (1984) Revisited
Source: Front Psychol. 2019 Oct 17;10:2227. doi: 10.3389/fpsyg.2019.02227 (PMC6811658; doi:10.3389/fpsyg.2019.02227)
Supplement: Supplementary file 1 [file Table_1.DOCX]

Supplementary Material 0. References of the technical reports included in the meta-analyses. All the technical reports can be downloaded from [www.eric.edu.gov](http://www.eric.edu.gov) using the eric id code (e.g., ED059285)

*USES (1950). Technical report on standardization of the General Aptitude Test Battery for Bookkeeping Machine Operator 1-02.01. Washington, DC: U.S. Department of Labor, Bureau of Employment Security. Available from Eric ED059285.

*USES (1951). Technical report on standardization of the General Aptitude Test Battery for Bomb-Fuse Parts Assembler (Ammunition), 6-54.052. Washington, DC: U.S. Department of Labor, Bureau of Employment Security. Available from Eric ED059300.

*USES (1951). Technical report on standardization of the General Aptitude Test Battery for Bomb-Fuse Parts Assembler (Ammunition), 6-54.052. Washington, DC: U.S. Department of Labor, Bureau of Employment Security. Available from Eric ED059300.

*USES (1951). Technical report on standardization of the General Aptitude Test Battery for Casing Tier 529.887.020. Washington, DC: U.S. Department of Labor, Bureau of Employment Security. Available from Eric ED060075.

*USES (1952). Technical report on standardization of the General Aptitude Test Battery for Sewer, Hand II, 6-25.050. Washington, DC: U.S. Department of Labor, Bureau of Employment Security. Available from Eric ED059301.

*USES (1952). Technical report on standardization of the General Aptitude Test Battery for Laborer, Poultry, 6-09.01. Washington, DC: U.S. Department of Labor, Bureau of Employment Security. Available from Eric ED059302.

*USES (1952). Technical report on standardization of the General Aptitude Test Battery for Packer, Tea Bag, 9-68.01. Washington, DC: U.S. Department of Labor, Bureau of Employment Security. Available from Eric ED059305.

*USES (1952). Technical report on standardization of the General Aptitude Test Battery for Manager, Theater 0-98.54. Washington, DC: U.S. Department of Labor, Bureau of Employment Security. Available from Eric ED060067.

*USES (1952). Technical report on standardization of the General Aptitude Test Battery for Turret Lathe Operator, 604.782. Washington, DC: U.S. Department of Labor, Bureau of Employment Security. Available from Eric ED060072.

*USES (1952). Technical report on standardization of the General Aptitude Test Battery for Turret Lathe Operator, 4-78.021. Washington, DC: U.S. Department of Labor, Bureau of Employment Security. Available from Eric ED060072.

*USES (1953). Technical report on standardization of the General Aptitude Test Battery for File Clerk II, 1-17.02. Washington, DC: U.S. Department of Labor, Bureau of Employment Security. Available from Eric ED060078 (Erroneous Code ED060073).

*USES (1953). Technical report on standardization of the General Aptitude Test Battery for General Practitioner, 070.103. Washington, DC: U.S. Department of Labor, Bureau of Employment Security. Available from Eric ED060079.

*USES (1953). Technical report on standardization of the General Aptitude Test Battery for Boarder II, 6-14.171. Washington, DC: U.S. Department of Labor, Bureau of Employment Security. Available from Eric ED060081.

*USES (1953). Technical report on standardization of the General Aptitude Test Battery for Tabulating Machine Operator, 213.782. Washington, DC: U.S. Department of Labor, Bureau of Employment Security. Available from Eric ED060082.

*USES (1953). Technical report on standardization of the General Aptitude Test Battery for Forming-Press Operator, 6-88.627. Washington, DC: U.S. Department of Labor, Bureau of Employment Security. Available from Eric ED060084.

*USES (1954). Technical report on standardization of the General Aptitude Test Battery for Stock Clerk II 1-38.01. Washington, DC: U.S. Department of Labor, Bureau of Employment Security. Available from Eric 059282.

*USES (1954). Technical report on standardization of the General Aptitude Test Battery for Laborer. Washington, DC: U.S. Department of Labor, Bureau of Employment Security. Available from Eric 059283.

*USES (1954). Technical report on standardization of the General Aptitude Test Battery for Wrapper-Layer Examiner, 6-12.341. Washington, DC: U.S. Department of Labor, Bureau of Employment Security. Available from Eric ED059297.

*USES (1954). Technical report on standardization of the General Aptitude Test Battery for Electric Motor Assembler, 6-99.166. Washington, DC: U.S. Department of Labor, Bureau of Employment Security. Available from Eric ED059298.

*USES (1954). Technical report on standardization of the General Aptitude Test Battery for Table Worker 8-53.01. Washington, DC: U.S. Department of Labor, Bureau of Employment Security. Available from Eric ED059304.

*USES (1954). Technical report on standardization of the General Aptitude Test Battery for Teacher, Nursery School, 359.878. Washington, DC: U.S. Department of Labor, Bureau of Employment Security. Available from Eric ED060087.

*USES (1954). Technical report on standardization of the General Aptitude Test Battery for Dentist, 072-108. Washington, DC: U.S. Department of Labor, Bureau of Employment Security. Available from Eric ED060088.

*USES (1954). Technical report on standardization of the General Aptitude Test Battery for Carding Machine Operator, 8-27.77. Washington, DC: U.S. Department of Labor, Bureau of Employment Security. Available from Eric ED060089.

*USES (1954). Technical report on standardization of the General Aptitude Test Battery for Fireworks Assembler, 737.887. Washington, DC: U.S. Department of Labor, Bureau of Employment Security. Available from Eric ED060091.

*USES (1954). Technical report on standardization of the General Aptitude Test Battery for Spinner, Ring Frame, 682.885. Washington, DC: U.S. Department of Labor, Bureau of Employment Security. Available from Eric ED060092.

*USES (1954). Technical report on standardization of the General Aptitude Test Battery for Dental Hygienist, 078.368. Washington, DC: U.S. Department of Labor, Bureau of Employment Security. Available from Eric ED060093.

*USES (1954). Technical report on standardization of the General Aptitude Test Battery for Dietitian, 0-39.93. Washington, DC: U.S. Department of Labor, Bureau of Employment Security. Available from Eric ED060094.

*USES (1954). Technical report on standardization of the General Aptitude Test Battery for Light-Bulb Assembler, 7-00.070. Washington, DC: U.S. Department of Labor, Bureau of Employment Security. Available from Eric ED060095.

*USES (1954). Technical report on standardization of the General Aptitude Test Battery for Furniture Upholsterer 780.381. Washington, DC: U.S. Department of Labor, Bureau of Employment Security. Available from Eric ED060096.

*USES (1954). Technical report on standardization of the General Aptitude Test Battery for Veterinarian 0-34.10. Washington, DC: U.S. Department of Labor, Bureau of Employment Security. Available from Eric ED060097.

*USES (1954). Technical report on standardization of the General Aptitude Test Battery for Dressmaker 4-25-030. Washington, DC: U.S. Department of Labor, Bureau of Employment Security. Available from Eric ED060098.

*USES (1954). Technical report on standardization of the General Aptitude Test Battery for Pairer (Hosiery) 6-14.341. Washington, DC: U.S. Department of Labor, Bureau of Employment Security. Available from Eric ED060099.

*USES (1955). Technical report on standardization of the General Aptitude Test Battery for Decorator, Hand 7-16.900. Washington, DC: U.S. Department of Labor, Bureau of Employment Security. Available from Eric ED059296.

*USES (1955). Technical report on standardization of the General Aptitude Test Battery for Fettler 8-66.01. Washington, DC: U.S. Department of Labor, Bureau of Employment Security. Available from Eric ED059299.

*USES (1955). Technical report on standardization of the General Aptitude Test Battery for Stripper, Hand (Tobacco) 3-12.10. Washington, DC: U.S. Department of Labor, Bureau of Employment Security. Available from Eric ED060101.

*USES (1955). Technical report on standardization of the General Aptitude Test Battery for Folder (Garment) III 6-27.988. Washington, DC: U.S. Department of Labor, Bureau of Employment Security. Available from Eric ED060102.

*USES (1955). Technical report on standardization of the General Aptitude Test Battery for Material Coordinator 221.168. Washington, DC: U.S. Department of Labor, Bureau of Employment Security. Available from Eric ED060103.

*USES (1955). Technical report on standardization of the General Aptitude Test Battery for Stocking Inspector I 6-14.235. Washington, DC: U.S. Department of Labor, Bureau of Employment Security. Available from Eric ED060104.

*USES (1955). Technical report on standardization of the General Aptitude Test Battery for Assembler, Dry Cell Battery 9-00.91. Washington, DC: U.S. Department of Labor, Bureau of Employment Security. Available from Eric ED060105.

*USES (1955). Technical report on standardization of the General Aptitude Test Battery for Stillman 542.280. Washington, DC: U.S. Department of Labor, Bureau of Employment Security. Available from Eric ED060107.

*USES (1955). Technical Report on the Development of the General Aptitude Test Battery for Artificial-Breeding Technician II, 467.364. Washington, DC: U.S. Department of Labor. Available from Eric ED060109.

*USES (1955). Technical report on standardization of the General Aptitude Test Battery for Mender, 782-884. Washington, DC: U.S. Department of Labor, Bureau of Employment Security. Available from Eric ED060114.

*USES (1956). Technical report on standardization of the General Aptitude Test Battery for Tomato Peeler, 529.887. Washington, DC: U.S. Department of Labor, Bureau of Employment Security. Available from Eric ED060116.

*USES (1956). Technical report on standardization of the General Aptitude Test Battery for Assembler, Electrical Accessories II 7-00.904. Washington, DC: U.S. Department of Labor, Bureau of Employment Security. Available from Eric ED060120.

*USES (1956). Technical report on standardization of the General Aptitude Test Battery for Scrapper 794.887. Washington, DC: U.S. Department of Labor, Bureau of Employment Security. Available from Eric ED060122.

*USES (1956). Technical report on standardization of the General Aptitude Test Battery for Take-Off Man 8-42.01. Washington, DC: U.S. Department of Labor, Bureau of Employment Security. Available from Eric ED060123.

*USES (1956). Technical report on standardization of the General Aptitude Test Battery for Multiple-Photographic Printer Operator 976.782. Washington, DC: U.S. Department of Labor, Bureau of Employment Security. Available from Eric ED060125.

*USES (1956). Technical report on standardization of the General Aptitude Test Battery for Clothes Designer, 142.081. Washington, DC: U.S. Department of Labor, Bureau of Employment Security. Available from Eric ED060127.

*USES (1956). Technical report on standardization of the General Aptitude Test Battery for Spot-Welder Feeder, 819.886. Washington, DC: U.S. Department of Labor, Bureau of Employment Security. Available from Eric ED060128.

*USES (1956). Technical report on standardization of the General Aptitude Test Battery for Calculating-Machine Operator, 1-25.13. Washington, DC: U.S. Department of Labor, Bureau of Employment Security. Available from Eric ED060129.

*USES (1956). Technical report on standardization of the General Aptitude Test Battery for Peeling-and-Coring-machine Operator 529.886. Washington, DC: U.S. Department of Labor, Bureau of Employment Security. Available from Eric ED061290.

*USES (1956). Technical report on standardization of the General Aptitude Test Battery for Director, Funeral 0-65.10. Washington, DC: U.S. Department of Labor, Bureau of Employment Security. Available from Eric ED061291.

*USES (1956). Technical report on standardization of the General Aptitude Test Battery for Bagger II, 9-68.01. Washington, DC: U.S. Department of Labor, Bureau of Employment Security. Available from Eric ED061293.

*USES (1956). Technical report on standardization of the General Aptitude Test Battery for Nut Sorter I, 8-10.25. Washington, DC: U.S. Department of Labor, Bureau of Employment Security. Available from Eric ED061301.

*USES (1957). Technical report on standardization of the General Aptitude Test Battery for Cementer, 8-57.51. Washington, DC: U.S. Department of Labor, Bureau of Employment Security. Available from Eric ED061292.

*USES (1957). Technical report on standardization of the General Aptitude Test Battery for Crusher Inspector, 631.381. Washington, DC: U.S. Department of Labor, Bureau of Employment Security. Available from Eric ED061302.

*USES (1957). Technical report on standardization of the General Aptitude Test Battery for Cold Mill Operator, 4-88.018. Washington, DC: U.S. Department of Labor, Bureau of Employment Security. Available from Eric ED061303.

*USES (1957). Technical report on standardization of the General Aptitude Test Battery for Candy-Wrapping-Machine Operator, 920.885.034. Washington, DC: U.S. Department of Labor, Bureau of Employment Security. Available from Eric ED061304.

*USES (1957). Technical Report on the Development of USTES Aptitude Test Battery for Assembler, Automobile, 806.887. Washington, DC: U.S. Department of Labor, Bureau of Employment Security. Available from Eric ED061306.

*USES (1957). Technical Report on the Development of USTES Aptitude Test Battery for Coil Assembler, 6-94.515. Washington, DC: U.S. Department of Labor, Bureau of Employment Security. Available from Eric ED061307.

*USES (1957). Technical report on standardization of the General Aptitude Test Battery for Employment Clerk 205.368. Washington, DC: U.S. Department of Labor, Bureau of Employment Security. Available from Eric ED061310.

*USES (1957). Technical report on standardization of the General Aptitude Test Battery for Power-Plant Operator I, 952.782. Washington, DC: U.S. Department of Labor, Bureau of Employment Security. Available from Eric ED061311.

*USES (1957). Technical report on standardization of the General Aptitude Test Battery for Copy Holder (Clerical), 209.588. Washington, DC: U.S. Department of Labor, Bureau of Employment Security. Available from Eric ED061313.

*USES (1957). Technical report on standardization of the General Aptitude Test Battery for Pantographer, 979.782. Washington, DC: U.S. Department of Labor, Bureau of Employment Security. Available from Eric ED061314.

*USES (1957). Technical report on standardization of the General Aptitude Test Battery for Paster 573.884. Washington, DC: U.S. Department of Labor, Bureau of Employment Security. Available from Eric ED061317.

*USES (1957). Technical report on standardization of the General Aptitude Test Battery for Clicking-Machine Operator 6-62.055. Washington, DC: U.S. Department of Labor, Bureau of Employment Security. Available from Eric ED061328.

*USES (1958). Technical report on standardization of the General Aptitude Test Battery for Fruit Sorter 9-68.60. Washington, DC: U.S. Department of Labor, Bureau of Employment Security. Available from Eric ED061321.

*USES (1958). Technical report on standardization of the General Aptitude Test Battery for Grid Operator 6-98.251. Washington, DC: U.S. Department of Labor, Bureau of Employment Security. Available from Eric ED061322.

*USES (1958). Technical report on standardization of the General Aptitude Test Battery for Egg Candler 7-76.110. Washington, DC: U.S. Department of Labor, Bureau of Employment Security. Available from Eric ED061323.

*USES (1958). Technical report on standardization of the General Aptitude Test Battery for Hosiery Looper 689.782. Washington, DC: U.S. Department of Labor, Bureau of Employment Security. Available from Eric ED061324.

*USES (1958). Technical report on standardization of the General Aptitude Test Battery for Corn-Cutting Machine Operator 529.886. Washington, DC: U.S. Department of Labor, Bureau of Employment Security. Available from Eric ED061326.

*USES (1958). Technical report on standardization of the General Aptitude Test Battery for Clicking-Machine Operator 6-62.055. Washington, DC: U.S. Department of Labor, Bureau of Employment Security. Available from Eric ED061328.

*USES (1958). Technical report on standardization of the General Aptitude Test Battery for Transfer Knitter 6-14.063. Washington, DC: U.S. Department of Labor, Bureau of Employment Security. Available from Eric ED061330.

*USES (1958). Technical report on standardization of the General Aptitude Test Battery for Cereal Packer 920.887. Washington, DC: U.S. Department of Labor, Bureau of Employment Security. Available from Eric ED061332.

*USES (1958). Technical report on standardization of the General Aptitude Test Battery for Seamless-Hosiery Knitter 684.885. Washington., DC: U.S. Department of Labor, Bureau of Employment Security. Available from Eric ED061334.

*USES (1958). Technical report on standardization of the General Aptitude Test Battery for Patternmaker 5-17.010. Washington., DC: U.S. Department of Labor, Bureau of Employment Security. Available from Eric ED061336.

*USES (1959). Technical report on standardization of the General Aptitude Test Battery for Firesetter 692.380. Washington., DC: U.S. Department of Labor, Bureau of Employment Security. Available from Eric ED061337.

*USES (1958). Technical report on standardization of the General Aptitude Test Battery for Luggage-Hardware Assembler 6-93.404. Washington, DC: U.S. Department of Labor, Bureau of Employment Security. Available from Eric ED061339.

*USES (1958). Technical report on standardization of the General Aptitude Test Battery for Waxed-Bag-Machine Operator 649.340. Washington, DC: U.S. Department of Labor, Bureau of Employment Security. Available from Eric ED061340.

*USES (1958). Technical report on standardization of the General Aptitude Test Battery for Presser 7-57.501. Washington, DC: U.S. Department of Labor, Bureau of Employment Security. Available from Eric ED061341.

*USES (1958). Technical report on standardization of the General Aptitude Test Battery for Water Filterer (waterworks) 7-54.621. Washington, DC: U.S. Department of Labor, Bureau of Employment Security. Available from Eric ED061342.

*USES (1958). Technical report on standardization of the General Aptitude Test Battery for Power Lawn Mower Assembler 6-94.352. Washington, DC: U.S. Department of Labor, Bureau of Employment Security. Available from Eric ED062405.

*USES (1958). Technical report on standardization of the General Aptitude Test Battery for Solderer I, 6-95.001. Washington, DC: U.S. Department of Labor, Bureau of Employment Security. Available from Eric ED062406.

*USES (1958). Technical report on standardization of the General Aptitude Test Battery for Jewelry Assembler 6-72.333. Washington, DC: U.S. Department of Labor, Bureau of Employment Security. Available from Eric ED062407.

*USES (1958). Technical report on standardization of the General Aptitude Test Battery for Woodworking Machine Operator 6-33.910 . Washington, DC: U.S. Department of Labor, Bureau of Employment Security. Available from Eric ED062408.

*USES (1959). Technical report on standardization of the General Aptitude Test Battery for Presser Machine 7-57.511. Washington, DC: U.S. Department of Labor, Bureau of Employment Security. Available from Eric ED062410.

*USES (1959). Technical report on standardization of the General Aptitude Test Battery for Cementer, Hand II, 6-61.311. Washington, DC: U.S. Department of Labor, Bureau of Employment Security. Available from Eric ED062411.

*USES (1959). Technical report on standardization of the General Aptitude Test Battery for Goggle Glass Cutter 713.884. Washington, DC: U.S. Department of Labor, Bureau of Employment Security. Available from Eric ED062412.

*USES (1959). Technical report on standardization of the General Aptitude Test Battery for Caser, 6-72.105. Washington, DC: U.S. Department of Labor, Bureau of Employment Security. Available from Eric ED062413.

*USES (1959). Technical report on standardization of the General Aptitude Test Battery for Loom Fixer, 4-16.010. Washington, DC: U.S. Department of Labor, Bureau of Employment Security. Available from Eric ED062417.

*USES (1959). Technical report on standardization of the General Aptitude Test Battery for Radio Receiver Assembler, 6-98.010. Washington, DC: U.S. Department of Labor, Bureau of Employment Security. Available from Eric ED062419.

*USES (1959). Technical report on standardization of the General Aptitude Test Battery for Medical Technologist 0-50.01. Washington, DC: U.S. Department of Labor, Bureau of Employment Security. Available from Eric ED062420.

*USES (1959). Technical report on standardization of the General Aptitude Test Battery for Manager, Industrial Organization, 0-97.41. Washington, DC: U.S. Department of Labor, Bureau of Employment Security. Available from Eric ED062422.

*USES (1959). Technical report on standardization of the General Aptitude Test Battery for Fancy Stitcher 690.782. Washington, DC: U.S. Department of Labor, Bureau of Employment Security. Available from Eric ED062423.

*USES (1959). Technical report on standardization of the General Aptitude Test Battery for Mail Sorter 1-27.10. Washington, DC: U.S. Department of Labor, Bureau of Employment Security. Available from Eric ED062426.

*USES (1959). Technical report on standardization of the General Aptitude Test Battery for Merchandise Packer 9-68.30. Washington, DC: U.S. Department of Labor, Bureau of Employment Security. Available from Eric ED062429.

*USES (1959). Technical report on standardization of the General Aptitude Test Battery for Die-Casting-Machine Operator II, 6-82.916. Washington, DC: U.S. Department of Labor, Bureau of Employment Security. Available from Eric ED062430.

*USES (1960). Technical report on standardization of the General Aptitude Test Battery for Chemist, Assistant 0-50.22. Washington, DC: U.S. Department of Labor, Bureau of Employment Security. Available from Eric ED059286.

*USES (1960). Technical report on standardization of the General Aptitude Test Battery for Seamer 6-27.513. Washington, DC: U.S. Department of Labor, Bureau of Employment Security. Available from Eric ED060117.

*USES (1960). Technical report on standardization of the General Aptitude Test Battery for Countergirl-Counterman, Assistant 2-27.13. Washington, DC: U.S. Department of Labor, Bureau of Employment Security. Available from Eric ED062428.

*USES (1960). Technical report on standardization of the General Aptitude Test Battery for Parts Storekeeper, 1-75.24. Washington, DC: U.S. Department of Labor, Bureau of Employment Security. Available from Eric ED062431.

*USES (1960). Technical report on standardization of the General Aptitude Test Battery for Coil Assembler, 6-99.161. Washington, DC: U.S. Department of Labor, Bureau of Employment Security. Available from Eric ED062432.

*USES (1960). Technical report on standardization of the General Aptitude Test Battery for Electronics-Unit Assembler, 6-98.604. Washington, DC: U.S. Department of Labor, Bureau of Employment Security. Available from Eric ED062436.

*USES (1961). Technical report on standardization of the General Aptitude Test Battery for Press Operator, 4-65.415. Washington, DC: U.S. Department of Labor, Bureau of Employment Security. Available from Eric ED062433.

*USES (1961). Technical report on standardization of the General Aptitude Test Battery for Folding-Machine Operator, 4-49.051. Washington, DC: U.S. Department of Labor, Bureau of Employment Security. Available from Eric ED062435.

*USES (1961). Technical report on standardization of the General Aptitude Test Battery for Case Coverer, 6-62.401. Washington, DC: U.S. Department of Labor, Bureau of Employment Security. Available from Eric ED062437.

*USES (1961). Technical report on standardization of the General Aptitude Test Battery for Case Worker, 0-27.20. Washington, DC: U.S. Department of Labor, Bureau of Employment Security. Available from Eric ED062438.

*USES (1961). Technical report on standardization of the General Aptitude Test Battery for Waitress, 2-27.12. Washington, DC: U.S. Department of Labor, Bureau of Employment Security. Available from Eric ED062442.

*USES (1961). Technical report on standardization of the General Aptitude Test Battery for Bookkeeper II, 1-01.02. Washington, DC: U.S. Department of Labor, Bureau of Employment Security. Available from Eric ED062446.

*USES (1962). Technical report on standardization of the General Aptitude Test Battery for Glass Products Inspector, School Lunch Program 0-71.32. Washington, DC: U.S. Department of Labor, Bureau of Employment Security. Available from Eric ED062441.

*USES (1962). Technical report on standardization of the General Aptitude Test Battery for Key-Punch Operator, 1-25.62. Washington, DC: U.S. Department of Labor, Bureau of Employment Security. Available from Eric ED062443.

*USES (1962). Technical report on standardization of the General Aptitude Test Battery for Service Engineer, 5-83.988. Washington, DC: U.S. Department of Labor, Bureau of Employment Security. Available from Eric ED062445.

*USES (1962). Technical report on standardization of the General Aptitude Test Battery for Carpet Layer, 5-32.732. Washington, DC: U.S. Department of Labor, Bureau of Employment Security. Available from Eric ED062447.

*USES (1962). Technical report on standardization of the General Aptitude Test Battery for Candy Packer, 8-05.21. Washington, DC: U.S. Department of Labor, Bureau of Employment Security. Available from Eric ED062448.

*USES (1962). Technical report on standardization of the General Aptitude Test Battery for Metal-Chair Assembler II, 6-36.275. Washington, DC: U.S. Department of Labor, Bureau of Employment Security. Available from Eric ED063380.

*USES (1962). Technical report on standardization of the General Aptitude Test Battery for Routeman, Retail Dairy Products, 1-80.06. Washington, DC: U.S. Department of Labor, Bureau of Employment Security. Available from Eric ED063381.

*USES (1962). Technical report on standardization of the General Aptitude Test Battery for Routeman, Wholesale Dairy Products, 1-80.06. Washington, DC: U.S. Department of Labor, Bureau of Employment Security. Available from Eric ED063382.

*USES (1962). Technical report on standardization of the General Aptitude Test Battery for Routeman, Bakery Products, 1-80.06. Washington, DC: U.S. Department of Labor, Bureau of Employment Security. Available from Eric ED063383.

*USES (1962). Technical report on standardization of the General Aptitude Test Battery for Automobile-Service-Station Attendant, 7-60.500. Washington, DC: U.S. Department of Labor, Bureau of Employment Security. Available from Eric ED063384.

*USES (1962). Technical report on standardization of the General Aptitude Test Battery for Audit Clerk, 1-01.32. Washington, DC: U.S. Department of Labor, Bureau of Employment Security. Available from Eric ED063385.

*USES (1962). Technical report on standardization of the General Aptitude Test Battery for Ticket Agent, 1-44.12. Washington, DC: U.S. Department of Labor, Bureau of Employment Security. Available from Eric ED063386.

*USES (1962). Technical report on standardization of the General Aptitude Test Battery for Painter-Decorator, 5-27.010. Washington, DC: U.S. Department of Labor, Bureau of Employment Security. Available from Eric ED063389.

*USES (1962). Technical report on standardization of the General Aptitude Test Battery for Director, School Lunch, 0-71.32. Washington, DC: U.S. Department of Labor, Bureau of Employment Security. Available from Eric ED063390.

*USES (1962). Technical report on standardization of the General Aptitude Test Battery for Occupational Analyst, 0-39.85. Washington, DC: U.S. Department of Labor, Bureau of Employment Security. Available from Eric ED063391.

*USES (1962). Technical report on standardization of the General Aptitude Test Battery for Telephone Deputy, 1-18.44. Washington, DC: U.S. Department of Labor, Bureau of Employment Security. Available from Eric ED063392.

*USES (1962). Technical report on standardization of the General Aptitude Test Battery Welder, Inert Gas, 4-85.025. Washington, DC: U.S. Department of Labor, Bureau of Employment Security. Available from Eric ED063393.

*USES (1962). Technical report on standardization of the General Aptitude Test Battery for Gas Serviceman, 5-83.947. Washington, DC: U.S. Department of Labor, Bureau of Employment Security. Available from Eric ED063394.

*USES (1962). Technical report on standardization of the General Aptitude Test Battery for Wire Drawer, 4-88.511. Washington, DC: U.S. Department of Labor, Bureau of Employment Security. Available from Eric ED063400.

*USES (1962). Technical report on standardization of the General Aptitude Test Battery for Autoclave Operator, 4-52.711. Washington, DC: U.S. Department of Labor, Bureau of Employment Security. Available from Eric ED065574.

*USES (1963). Technical report on standardization of the General Aptitude Test Battery for Salesman, Construction Machinery, 1-86.26. Washington, DC: U.S. Department of Labor, Bureau of Employment Security. Available from Eric ED063396.

*USES (1963). Technical report on standardization of the General Aptitude Test Battery for Mathematician, 020.088-118. Washington, DC: U.S. Department of Labor, Bureau of Employment Security. Available from Eric ED063399.

*USES (1963). Technical report on standardization of the General Aptitude Test Battery for Braiding-Machine Operator, 6-19.986. Washington, DC: U.S. Department of Labor, Bureau of Employment Security. Available from Eric ED063402.

*USES (1963). Technical report on standardization of the General Aptitude Test Battery for Condenser Winder, 9,68.20. Washington, DC: U.S. Department of Labor, Bureau of Employment Security. Available from Eric ED063404.

*USES (1963). Technical report on standardization of the General Aptitude Test Battery for Coil Winder II, 724.884. Washington, DC: U.S. Department of Labor, Bureau of Employment Security. Available from Eric ED063405.

*USES (1963). Technical report on standardization of the General Aptitude Test Battery for Extruder Operator, 6-51.468. Washington, DC: U.S. Department of Labor, Bureau of Employment Security. Available from Eric ED063406.

*USES (1963). Technical report on standardization of the General Aptitude Test Battery for Teller 212.368. Washington, DC: U.S. Department of Labor, Bureau of Employment Security. Available from Eric ED065583.

*USES (1963). Technical report on standardization of the General Aptitude Test Battery for Air Traffic Control Specialist, 0-61.60. Washington, DC: U.S. Department of Labor, Bureau of Employment Security. Available from Eric ED065580.

*USES (1963). Technical report on standardization of the General Aptitude Test Battery for Assembler, 9-57.21. Washington, DC: U.S. Department of Labor, Bureau of Employment Security. Available from Eric ED065606.

*USES (1963). Technical report on standardization of the General Aptitude Test Battery for Container Maker-Filler-Packer Operator, 7-68.920. Washington, DC: U.S. Department of Labor, Bureau of Employment Security. Available from Eric ED065566.

*USES (1963). Technical report on standardization of the General Aptitude Test Battery for Finisher I, 9-10.10. Washington, DC: U.S. Department of Labor, Bureau of Employment Security. Available from Eric ED065570.

*USES (1964). Technical report on standardization of the General Aptitude Test Battery for Teacher, Grade or Grammar School, 0-31.01. Washington, DC: U.S. Department of Labor, Manpower Administration. Available from Eric ED060126.

*USES (1965). Technical report on standardization of the General Aptitude Test Battery for Baser, 7-00.070. Washington, DC: U.S. Department of Labor, Bureau of Employment Security. Available from Eric ED059303.

*USES (1965). Technical report on standardization of the General Aptitude Test Battery for Tube-Machine Operator, 7-00.216. Washington, DC: U.S. Department of Labor, Bureau of Employment Security. Available from Eric ED069779.

*USES (1965). Technical report on standardization of the General Aptitude Test Battery for Experimental Assembler, 6-78.642. Washington, DC: U.S. Department of Labor, Bureau of Employment Security. Available from Eric ED069777.

*USES (1965). Technical report on standardization of the General Aptitude Test Battery for Card Tender, 6-19.031. Washington, DC: U.S. Department of Labor, Bureau of Employment Security. Available from Eric ED070772.

*USES (1966). Technical Report on the Development of USES Aptitude Test Battery for Printed-Napkin-Machine Operator 649.885. Washington, DC: U.S. Department of Labor, Bureau of Employment Security. Available from Eric ED060112.

*USES (1966). Technical Report on the Development of USES Aptitude Test Battery for Trailer-Tank-Truck Driver 903.883. Washington, DC: U.S. Department of Labor, Bureau of Employment Security. Available from Eric ED061329.

*USES (1966). Technical Report on the Development of USES Aptitude Test Battery for Production-Machine Operator 609.885. Washington, DC: U.S. Department of Labor, Bureau of Employment Security. Available from Eric ED061338.

*USES (1966). Technical report on standardization of the General Aptitude Test Battery for Order Filler, 922.887. Washington, DC: U.S. Department of Labor, Bureau of Employment Security. Available from Eric ED062444.

*USES (1966). Technical report on standardization of the General Aptitude Test Battery for Dental Assistant, 079.378. Washington, DC: U.S. Department of Labor, Bureau of Employment Security. Available from Eric ED063388.

*USES (1966). Technical report on standardization of the General Aptitude Test Battery for Welder, Arc, 810.884. Washington, DC: U.S. Department of Labor, Bureau of Employment Security. Available from Eric ED063397.

*USES (1966). Technical Report on the Development of USES Aptitude Test Battery for Asparagus Sorter 529.687. Washington, DC: U.S. Department of Labor, Bureau of Employment Security. Available from Eric ED069769.

*USES (1967). Technical report on standardization of the USES Aptitude Test Battery for Mounter 726.887. Washington, DC: U.S. Department of Labor, Bureau of Employment Security. Available from Eric ED059287.

*USES (1967). Technical report on standardization of the USES Aptitude Test Battery for Pharmacist, 074.181. Washington, DC: U.S. Department of Labor, Bureau of Employment Security. Available from Eric ED060077.

*USES (1967). Technical report on standardization of the USES Aptitude Test Battery for Radiologic Technologist 078.368. Washington, DC: U.S. Department of Labor, Manpower Administration. Available from Eric ED060119.

*USES (1967). Technical report on standardization of the USES Aptitude Test Battery for Accountant 160.188. Washington, DC: U.S. Department of Labor, Manpower Administration. Available from Eric ED061319.

*USES (1967). Technical report on standardization of the USES Aptitude Test Battery for Weaver, 683.782. Washington, DC: U.S. Department of Labor, Manpower Administration. Available from Eric ED061320.

*USES (1967). Technical report on standardization of the USES Aptitude Test Battery for Cannery Worker, 529.886. Washington, DC: U.S. Department of Labor, Manpower Administration. Available from Eric ED061327.

*USES (1967). Technical Report on the Development of USES Aptitude Test Battery for Counselor, Camp 159.228. Washington, DC: U.S. Department of Labor, Bureau of Employment Security. Available from Eric ED063395.

*USES (1968). Technical report on standardization of the General Aptitude Test Battery for Fishing-Rod Assembler 732.88. Washington, DC: U.S. Department of Labor, Manpower Administration. Available from Eric ED060118.

*USES (1968). Technical report on standardization of the General Aptitude Test Battery for Compression-Molding-Machine Tender, 556.885. Washington, DC: U.S. Department of Labor, Manpower Administration. Available from Eric ED063403.

*USES (1969). Technical report on standardization of the General Aptitude Test Battery for Stenographer 202.388. Washington, DC: U.S. Department of Labor, Manpower Administration. Available from Eric ED059288.

*USES (1969). Technical report on standardization of the General Aptitude Test Battery for Sewing Machine Operator, Selected 786.782 and 787.782. Washington, DC: U.S. Department of Labor, Manpower Administration. Available from Eric 059284.

*USES (1969). Technical Report on the Development of USTES Aptitude Test Battery for Photographer Finisher 976.886. Washington, DC: U.S. Department of Labor, Bureau of Employment Security. Available from Eric ED057168.

*USES (1969). Technical Report on the Development of USTES Aptitude Test Battery for Automobile Mechanic, 620.281. Washington, DC: U.S. Department of Labor, Bureau of Employment Security. Available from Eric ED060083.

*USES (1969). Technical Report on the Development of USTES Aptitude Test Battery for Molded-Goods Inspector-Trimmer 759.687. Washington, DC: U.S. Department of Labor, Bureau of Employment Security. Available from Eric ED069630.

*USES (1970). Technical report on standardization of the USTES Aptitude Test Battery for Carpenter 860.381. Washington, DC: U.S. Department of Labor, Manpower Administration. Available from Eric ED059289.

*USES (1970). Technical report on standardization of the USTES Aptitude Test Battery for Machinist 600.280. Washington, DC: U.S. Department of Labor, Manpower Administration. Available from Eric ED059290.

*USES (1970). Technical report on standardization of the USTES Aptitude Test Battery for Gasoline-Engine Assembler 806-701. Washington, DC: U.S. Department of Labor, Manpower Administration. Available from Eric ED059291.

*USES (1970). Technical report on standardization of the USTES Aptitude Test Battery for Pressman 559.885. Washington, DC: U.S. Department of Labor, Manpower Administration. Available from Eric ED059295.

*USES (1970). Technical Report on the Development of USTES Aptitude Test Battery for Bindery Worker 643.85. Washington, DC: U.S. Department of Labor, Manpower Administration. Available from Eric ED060074.

*USES (1970). Technical Report on the Development of USTES Aptitude Test Battery for Selected Press Man Occupations 651.782. Washington, DC: U.S. Department of Labor, Manpower Administration. Available from Eric ED060080.

*USES (1970). Technical Report on the Development of USTES Aptitude Test Battery for Shipfitter 806.381. Washington, DC: U.S. Department of Labor, Manpower Administration. Available from Eric ED060085.

*USES (1970). Technical Report on the Development of USTES Aptitude Test Battery for Compositor I, 973.381. Washington, DC: U.S. Department of Labor, Manpower Administration. Available from Eric ED060090.

*USES (1970). Technical Report on the Development of USTES Aptitude Test Battery for Pipe Fitter, 862.381. Washington, DC: U.S. Department of Labor, Manpower Administration. Available from Eric ED060100.

*USES (1970). Technical Report on the Development of USTES Aptitude Test Battery for Electrician, Airplane, 825.281. Washington, DC: U.S. Department of Labor, Manpower Administration. Available from Eric ED060108.

*USES (1970). Technical Report on the Development of USTES Aptitude Test Battery for Cosmetologist, 332.271. Washington, DC: U.S. Department of Labor, Manpower Administration. Available from Eric ED060110.

*USES (1970). Technical Report on the Development of USTES Aptitude Test Battery for Electrician, 824.281. Washington, DC: U.S. Department of Labor, Manpower Administration. Available from Eric ED060111.

*USES (1970). Technical Report on the Development of USTES Aptitude Test Battery for Central-Office Repairman, 822.281. Washington, DC: U.S. Department of Labor, Manpower Administration. Available from Eric ED060113.

*USES (1970). Technical Report on the Development of USTES Aptitude Test Battery for Selected Aircraft Assembly Occupations, 862.381. Washington, DC: U.S. Department of Labor, Manpower Administration. Available from Eric ED060115.

*USES (1970). Technical Report on the Development of USTES Aptitude Test Battery for Sheet-Metal Worker, 804.281. Washington, DC: U.S. Department of Labor, Manpower Administration. Available from Eric ED060121.

*USES (1970). Technical Report on the Development of USTES Aptitude Test Battery for Baker, 526.781. Washington, DC: U.S. Department of Labor, Manpower Administration. Available from Eric ED060124.

*USES (1970). Technical Report on the Development of USTES Aptitude Test Battery for Knitting-Machine Fixer, 689.280. Washington, DC: U.S. Department of Labor, Manpower Administration. Available from Eric ED061289.

*USES (1970). Technical Report on the Development of USTES Aptitude Test Battery for Cabinetmaker, 660.280. Washington, DC: U.S. Department of Labor, Manpower Administration. Available from Eric ED061305.

*USES (1970). Technical Report on the Development of USTES Aptitude Test Battery for Electronics Mechanic, 726.281. Washington, DC: U.S. Department of Labor, Manpower Administration. Available from Eric ED061308.

*USES (1970). Technical Report on the Development of USTES Aptitude Test Battery for Bricklayer, 861.381. Washington, DC: U.S. Department of Labor, Manpower Administration. Available from Eric ED061312.

*USES (1970). Technical Report on the Development of USTES Aptitude Test Battery for Aircraft-and-Engine Mechanic, 621.281. Washington, DC: U.S. Department of Labor, Manpower Administration. Available from Eric ED061316.

*USES (1970). Technical Report on the Development of USTES Aptitude Test Battery for Radio Repairman, 720.281. Washington, DC: U.S. Department of Labor, Manpower Administration. Available from Eric ED061318.

*USES (1970). Technical Report on the Development of USTES Aptitude Test Battery for Welder Combination, 812.884. Washington, DC: U.S. Department of Labor, Manpower Administration. Available from Eric ED061331.

*USES (1970). Technical Report on the Development of USTES Aptitude Test Battery for Linotype Operator, 650.582. Washington, DC: U.S. Department of Labor, Manpower Administration. Available from Eric ED061333.

*USES (1970). Technical Report on the Development of USTES Aptitude Test Battery for Stripper, 971.381. Washington, DC: U.S. Department of Labor, Manpower Administration. Available from Eric ED062404.

*USES (1970). Technical Report on the Development of USTES Aptitude Test Battery for Lineman Repair, 821.381. Washington, DC: U.S. Department of Labor, Manpower Administration. Available from Eric ED062418.

*USES (1970). Technical Report on the Development of USTES Aptitude Test Battery for Packaging-Machine Mechanic, 920.280. Washington, DC: U.S. Department of Labor, Manpower Administration. Available from Eric ED062421.

*USES (1970). Technical Report on the Development of USTES Aptitude Test Battery for Cable Maker, 726.884. Washington, DC: U.S. Department of Labor, Manpower Administration. Available from Eric ED062424.

*USES (1970). Technical Report on the Development of USTES Aptitude Test Battery for Monotype-Keyboard Operator, 650.582. Washington, DC: U.S. Department of Labor, Manpower Administration. Available from Eric ED062427.

*USES (1970). Technical Report on the Development of USTES Aptitude Test Battery for Glazier, 865.781. Washington, DC: U.S. Department of Labor, Manpower Administration. Available from Eric ED062434.

*USES (1970). Technical Report on the Development of USTES Aptitude Test Battery for Stereotyper, 975.782. Washington, DC: U.S. Department of Labor, Manpower Administration. Available from Eric ED062439.

*USES (1970). Technical Report on the Development of USTES Aptitude Test Battery for Manufacturers’ Service Representative, 638.281. Washington, DC: U.S. Department of Labor, Manpower Administration. Available from Eric ED062440.

*USES (1970). Technical Report on the Development of USTES Aptitude Test Battery for Construction-Equipment Mechanic, 620.281. Washington, DC: U.S. Department of Labor, Manpower Administration. Available from Eric ED063387.

*USES (1970). Technical Report on the Development of USTES Aptitude Test Battery for Tool-and-Die Maker, 601.280. Washington, DC: U.S. Department of Labor, Manpower Administration. Available from Eric ED063398.

*USES (1970). Technical Report on the Development of USTES Aptitude Test Battery for Cement Mason, 844.844. Washington, DC: U.S. Department of Labor, Manpower Administration. Available from Eric ED063401.

*USES (1970). Technical Report on the Development of USTES Aptitude Test Battery for Correction Officer 372.868. Washington, DC: U.S. Department of Labor, Manpower Administration. Available from Eric ED072051.

*USES (1971). Technical report on standardization of the USTES Aptitude Test Battery for Court Clerk 249.368. Washington, DC: U.S. Department of Labor, Bureau of Employment Security. Available from Eric ED060069.

*USES (1971). Technical report on standardization of the USTES Aptitude Test Battery for Engineer. Washington, DC: U.S. Department of Labor, Bureau of Employment Security. Available from Eric ED060076.

*USES (1971). Technical report on standardization of the USTES Aptitude Test Battery for Fork-Lift-Truck Operator. Washington, DC: U.S. Department of Labor, Bureau of Employment Security. Available from Eric ED061335.

*USES (1972). Technical Report on the Development of USTES Aptitude Test Battery for Taper 842.884. Washington, DC: U.S. Department of Labor, Bureau of Employment Security. Available from Eric ED072062.

*USES (1982). Technical Report on the Development of USES Specific Aptitude Test Battery for Carpenter 860.381-022. Washington, DC: U.S. Department of Labor, Bureau of Employment Security. Available from Eric ED223695.

*USES (1982). Technical Report on the Development of USES Specific Aptitude Test Battery for Ticket Agent 238.367-026. Washington, DC: U.S. Department of Labor, Bureau of Employment Security. Available from Eric ED223718.

*USES (19XX). Technical report on standardization of the General Aptitude Test Battery for Police 2-66.23. Washington, DC: U.S. Department of Labor, Bureau of Employment Security. Available from Eric ED065560.

Supplementary Material 1. Validity Coefficients and Range Restriction Values of G and GVN Composites for Grades.

| ERIC ID | Job Cplx | Sample | *r_g_* | *r_gvn_* | *u_gvn_* | *u_g_* |
| --- | --- | --- | --- | --- | --- | --- |
| ED059288 | 2 | 51 | .60 | .59 | .66 | .72 |
| ED059289 | 2 | 119 | .39 | .40 | .84 | .79 |
| ED060076 | 3 | 51 | .02 | .04 | .65 | .62 |
| ED060076 | 3 | 92 | .52 | .44 | - | - |
| ED060076 | 3 | 71 | .40 | .40 | - | - |
| ED060076 | 3 | 150 | .42 | .43 | .68 | .64 |
| ED060077 | 3 | 64 | .22 | .18 | .52 | .48 |
| ED060079 | 3 | 49 | .51 | .49 | .68 | .61 |
| ED060087 | 2 | 83 | .46 | .44 | .66 | .61 |
| ED060088 | 3 | 96 | .20 | .14 | .56 | .46 |
| ED060088 | 3 | 81 | .03 | -.08 | .70 | .69 |
| ED060093 | 2 | 83 | .48 | .36 | .59 | .54 |
| ED060097 | 3 | 33 | .30 | .28 | .67 | .62 |
| ED060097 | 3 | 37 | .31 | .30 | .57 | .52 |
| ED060098 | 2 | 55 | .21 | .11 | .60 | .50 |
| ED060119 | 2 | 40 | .46 | .48 | .76 | .76 |
| ED060121 | 2 | 79 | .47 | .44 | .74 | .70 |
| ED060124 | 2 | 65 | .35 | .26 | .65 | .61 |
| ED060127 | 3 | 102 | .37 | .36 | .62 | .56 |
| ED060127 | 3 | 47 | .16 | .11 | .75 | .67 |
| ED061291 | 3 | 50 | .47 | .52 | .63 | .62 |
| ED061311 | 2 | 54 | .45 | .39 | .58 | .57 |
| ED061312 | 2 | 50 | .38 | .43 | .62 | .56 |
| ED061316 | 2 | 75 | .41 | .40 | .65 | .70 |
| ED061318 | 2 | 62 | .40 | .32 | .54 | .51 |
| ED061318 | 2 | 30 | .33 | .30 | .68 | .67 |
| ED061318 | 2 | 35 | .24 | .28 | .68 | .77 |
| ED061319 | 3 | 30 | .51 | .53 | .70 | .67 |
| ED062422 | 3 | 70 | .52 | .43 | .77 | .78 |
| ED063389 | 2 | 49 | .40 | .37 | - | - |
| ED063398 | 2 | 59 | .48 | .40 | .73 | .75 |
| ED063399 | 3 | 52 | .29 | .28 | .78 | .71 |
| ED065561 | 2 | 51 | .62 | .68 | .77 | .73 |
| ED065562 | 2 | 160 | .22 | .16 | .72 | .68 |
| ED065578 | 3 | 51 | .41 | .30 | .69 | .61 |
| ED065612 | 2 | 52 | .32 | .28 | .50 | .48 |
| ED065612 | 2 | 63 | .37 | .37 | - | - |
| ED065616 | 2 | 111 | .46 | .46 | .66 | .62 |
| ED065617 | 3 | 80 | .40 | .34 | .65 | .62 |
| ED065617 | 3 | 94 | .49 | .48 | .68 | .65 |
| ED065617 | 3 | 100 | .43 | .43 | .58 | .59 |
| ED065618 | 2 | 65 | .39 | .31 | .70 | .66 |
| ED065629 | 3 | 50 | .49 | .44 | .60 | .57 |
| ED066461 | 3 | 85 | .33 | .42 | .66 | .62 |
| ED066480 | 2 | 65 | .42 | .39 | .75 | .76 |
| ED066483 | 3 | 55 | .21 | .22 | .59 | .56 |
| ED066484 | 3 | 63 | .39 | .37 | .59 | .58 |
| ED066485 | 3 | 52 | .11 | .19 | .53 | .53 |
| ED066486 | 3 | 59 | .36 | .27 | .53 | .54 |
| ED066487 | 3 | 55 | .46 | .33 | .65 | .63 |
| ED066489 | 2 | 73 | .43 | .47 | .69 | .69 |
| ED066509 | 2 | 54 | .39 | .33 | .63 | .55 |
| ED066513 | 2 | 80 | .44 | .40 | .67 | .62 |
| ED066514 | 3 | 51 | .13 | .19 | .60 | .67 |
| ED067419 | 2 | 81 | .56 | .54 | .63 | .63 |
| ED068544 | 2 | 66 | .53 | .54 | .63 | .64 |
| ED068546 | 3 | 241 | .45 | .43 | .65 | .65 |
| ED068552 | 3 | 70 | .16 | .18 | .57 | .58 |
| ED068554 | 2 | 52 | .40 | .38 | .62 | .61 |
| ED068566 | 3 | 52 | .19 | .15 | .61 | .59 |
| ED069627 | 3 | 50 | .10 | .13 | .50 | .45 |
| ED069633 | 3 | 64 | .58 | .55 | .60 | .61 |
| ED069642 | 2 | 61 | .24 | .20 | .78 | .72 |
| ED069768 | 2 | 57 | .40 | .39 | .68 | .64 |
| ED069772 | 2 | 105 | .36 | .37 | .67 | .66 |
| ED069773 | 3 | 55 | .37 | .40 | .67 | .66 |
| ED072048 | 2 | 50 | -.08 | -.11 | .57 | .61 |
| ED072050 | 3 | 122 | .37 | .33 | .67 | .63 |
| ED072050 | 3 | 102 | .26 | .28 | .64 | .60 |
| ED072055 | 3 | 84 | .20 | .24 | .64 | .57 |
|  |  |  |  |  |  |  |

Note. Job Cplx= job complexity 1= low level, 2 = medium level, 3 = high level; *r_g_ =* observed validity of G; *r_gvn_* = observed validity of GVN; *u_g_* = range restriction value of G; *u_gvn_* = range restriction value of GVN.

Supplementary Material 2. Validity Coefficients and Range Restriction Values of G and GVN Composites for Instructor Ratings.

| ERIC ID | Job Cplx | Sample | *r_g_* | *r_gvn_* | *u_gvn_* | *u_g_* |
| --- | --- | --- | --- | --- | --- | --- |
| ED059290 | 2 | 40 | .60 | .62 | .90 | .86 |
| ED060094 | 3 | 57 | .62 | .57 | .70 | .68 |
| ED060110 | 2 | 99 | .33 | .32 | .73 | .72 |
| ED060111 | 2 | 124 | .48 | .44 | .72 | .69 |
| ED060119 | 2 | 40 | .47 | .52 | .76 | .76 |
| ED061305 | 2 | 81 | .42 | .32 | .66 | .64 |
| ED061331 | 2 | 33 | .11 | .12 | .84 | .82 |
| ED061331 | 2 | 51 | .38 | .32 | - | - |
| ED061331 | 2 | 52 | .26 | .24 | .76 | .75 |
| ED061333 | 1 | 164 | .33 | .32 | .70 | .66 |
| ED061338 | 1 | 52 | .62 | - | - | .86 |
| ED062446 | 2 | 66 | .48 | .46 | .83 | .85 |
| ED063388 | 2 | 53 | .39 | .34 | .60 | .55 |
| ED063388 | 2 | 85 | .37 | .37 | .72 | .74 |
| ED063388 | 2 | 121 | .57 | .46 | .64 | .62 |
| ED063389 | 2 | 70 | .46 | .40 | .75 | .74 |
| ED063389 | 2 | 83 | .39 | .36 | - | - |
| ED065564 | 2 | 66 | .53 | .47 | .78 | .76 |
| ED065565 | 2 | 50 | .54 | .57 | .79 | .76 |
| ED065576 | 2 | 51 | .50 | .49 | .78 | .78 |
| ED065612 | 2 | 130 | .41 | .36 | .56 | .48 |
| ED065626 | 2 | 77 | .21 | .24 | .83 | .83 |
| ED066475 | 2 | 56 | .09 | .06 | .80 | .79 |
| ED066475 | 2 | 63 | .17 | .10 | .75 | .72 |
| ED066480 | 2 | 50 | .26 | .25 | .69 | .69 |
| ED066490 | 1 | 53 | .28 | .24 | .78 | .75 |
| ED066517 | 1 | 48 | .45 | .43 | .9 | .94 |
| ED067409 | 2 | 59 | .61 | .63 | .76 | .74 |
| ED068557 | 2 | 50 | .29 | .29 | .78 | .72 |
| ED068567 | 1 | 49 | .55 | .53 | .65 | .70 |
| ED069625 | 2 | 78 | .29 | .28 | .58 | .52 |
| ED069635 | 2 | 62 | .34 | .25 | .76 | .79 |
| ED069636 | 2 | 46 | .35 | .31 | .66 | .65 |
| ED069637 | 2 | 60 | .52 | .44 | .87 | .87 |
| ED069642 | 2 | 61 | .24 | .20 | .87 | .87 |
| ED070776 | 2 | 30 | .22 | .12 | .78 | .79 |
| ED070776 | 2 | 27 | .08 | .24 | .73 | .71 |
| ED072047 | 2 | 50 | .54 | .46 | .73 | .74 |
| ED072049 | 1 | 64 | .25 | .19 | .86 | .84 |
| ED072049 | 1 | 39 | .22 | .24 | .77 | .78 |
| ED072053 | 2 | 86 | .33 | .38 | .82 | .79 |
| ED078069 | 3 | 51 | .15 | .16 | .69 | .69 |
| ED118597 | 1 | 136 | .36 | .34 | .69 | .67 |
| ED158039 | 2 | 253 | .54 | .55 | .80 | .83 |
| ED223707 | 2 | 933 | .20 | .21 | .73 | .72 |
|  |  |  |  |  |  |  |

Note. Job Cplx= job complexity 1= low level, 2 = medium level, 3 = high level; *r_g_ =* observed validity of G; *r_gvn_* = observed validity of GVN; *u_g_* = range restriction value of G; *u_gvn_* = range restriction value of GVN.

Supplementary Material 3. Validity Coefficients and Range Restriction Values of G and GVN Composites for Job Performance Ratings.

| ERIC ID | Job Cplx | Sample | *r_g_* | *r_gvn_* | *u_gvn_* | *u_g_* |
| --- | --- | --- | --- | --- | --- | --- |
|  |  |  |  |  |  |  |
| ED016119 | 3 | 34 | .21 | .20 | .72 | .64 |
| ED016119 | 3 | 56 | .24 | .24 | .76 | .81 |
| ED057168 | 2 | 59 | .31 | .31 | .95 | .92 |
| ED059282 | 2 | 31 | .19 | .19 | .68 | .67 |
| ED059283 | 1 | 32 | .26 | .22 | .94 | .86 |
| ED059284 | 1 | 36 | .45 | .53 | - | - |
| ED059284 | 1 | 32 | .36 | .24 | - | - |
| ED059284 | 1 | 32 | .23 | .13 | - | - |
| ED059284 | 1 | 27 | .42 | .25 | - | - |
| ED059284 | 1 | 29 | .36 | .36 | - | - |
| ED059285 | 2 | 42 | .18 | .03 | .61 | .56 |
| ED059286 | 3 | 82 | .22 | .08 | .81 | .80 |
| ED059286 | 3 | 36 | .52 | .41 | .57 | .56 |
| ED059287 | 1 | 43 | .26 | .27 | - | - |
| ED059288 | 2 | 51 | .17 | .20 | .70 | .65 |
| ED059290 | 2 | 71 | .29 | .23 | .76 | .78 |
| ED059290 | 2 | 66 | .42 | - | - | .65 |
| ED059291 | 1 | 45 | .30 | .27 | .79 | .79 |
| ED059295 | 2 | 64 | .12 | .08 | .81 | .77 |
| ED059295 | 2 | 30 | .28 | .25 | .89 | .84 |
| ED059296 | 1 | 70 | .14 | .11 | .77 | .72 |
| ED059297 | 1 | 45 | .28 | .20 | .74 | .73 |
| ED059298 | 1 | 60 | .18 | .19 | 1.01 | .98 |
| ED059299 | 1 | 35 | .02 | .04 | .80 | .73 |
| ED059300 | 2 | 90 | .21 | .29 | .68 | .66 |
| ED059301 | 1 | 33 | .15 | .15 | .98 | .95 |
| ED059302 | 1 | 72 | .38 | .25 | .97 | .90 |
| ED059304 | 1 | 46 | -.19 | -.22 | .68 | .64 |
| ED059305 | 1 | 57 | .20 | .13 | .68 | .59 |
| ED060067 | 3 | 32 | .31 | .30 | .71 | .67 |
| ED060069 | 2 | 51 | .25 | .16 | .59 | .60 |
| ED060073 | 2 | 37 | .19 | .10 | .86 | .86 |
| ED060074 | 1 | 103 | .20 | .17 | .79 | .81 |
| ED060075 | 1 | 50 | .13 | .13 | .83 | .78 |
| ED060076 | 3 | 14 | .64 | .53 | .63 | .62 |
| ED060076 | 3 | 29 | .21 | .17 | - | - |
| ED060076 | 3 | 17 | .61 | .54 | - | - |
| ED060078 | 2 | 50 | .28 | .33 | .90 | .79 |
| ED060080 | 2 | 112 | .26 | .22 | .75 | .75 |
| ED060080 | 2 | 48 | .47 | .52 | .84 | .87 |
| ED060080 | 2 | 32 | .46 | .47 | .80 | .82 |
| ED060080 | 2 | 50 | .36 | .37 | .80 | .76 |
| ED060080 | 2 | 51 | .45 | .38 | .67 | .61 |
| ED060082 | 2 | 206 | .34 | .33 | .77 | .74 |
| ED060083 | 2 | 247 | .26 | .26 | .87 | .88 |
| ED060083 | 2 | 129 | .02 | .02 | .87 | .92 |
| ED060084 | 1 | 52 | .24 | .28 | .73 | .69 |
| ED060085 | 2 | 62 | .26 | .13 | .72 | .68 |
| ED060089 | 1 | 51 | .24 | .24 | .78 | .73 |
| ED060090 | 2 | 107 | .35 | .38 | .79 | .81 |
| ED060091 | 1 | 75 | .39 | .36 | .88 | .80 |
| ED060092 | 1 | 60 | .10 | .13 | .69 | .66 |
| ED060095 | 1 | 50 | .09 | .12 | .73 | .70 |
| ED060096 | 2 | 49 | .24 | .12 | .84 | .78 |
| ED060100 | 2 | 322 | .37 | .35 | .87 | .84 |
| ED060100 | 2 | 89 | .22 | .22 | .87 | .83 |
| ED060102 | 1 | 55 | .32 | .29 | .92 | .86 |
| ED060103 | 3 | 51 | .35 | .33 | .85 | .80 |
| ED060105 | 2 | 94 | .11 | .15 | .73 | .69 |
| ED060107 | 2 | 63 | .39 | .33 | .84 | .83 |
| ED060108 | 2 | 51 | .34 | .38 | .88 | .87 |
| ED060109 | 2 | 59 | .40 | .36 | .68 | .64 |
| ED060111 | 2 | 129 | .32 | .31 | .78 | .74 |
| ED060112 | 1 | 55 | .36 | .33 | .79 | .82 |
| ED060113 | 2 | 64 | .37 | .34 | .80 | .84 |
| ED060114 | 1 | 52 | .17 | .14 | .61 | .51 |
| ED060115 | 2 | 52 | .40 | .33 | .88 | .84 |
| ED060116 | 1 | 61 | .11 | .14 | .58 | .54 |
| ED060118 | 1 | 56 | .40 | .41 | .84 | .82 |
| ED060119 | 2 | 76 | .30 | .36 | .74 | .71 |
| ED060119 | 2 | 62 | .57 | .59 | .85 | .85 |
| ED060119 | 2 | 50 | .21 | .13 | .64 | .61 |
| ED060120 | 1 | 57 | .16 | .14 | .68 | .64 |
| ED060122 | 1 | 53 | .16 | .14 | .84 | .80 |
| ED060123 | 2 | 52 | .35 | .36 | .82 | .87 |
| ED060125 | 2 | 50 | .26 | .36 | .91 | .83 |
| ED060129 | 2 | 53 | -.06 | -.06 | .68 | .70 |
| ED060491 | 2 | 89 | .41 | .42 | .67 | .61 |
| ED061289 | 2 | 35 | .33 | .32 | .78 | .78 |
| ED061292 | 1 | 62 | .38 | .37 | .88 | .81 |
| ED061293 | 1 | 50 | .10 | .04 | .82 | .76 |
| ED061301 | 1 | 74 | -.09 | -.06 | .66 | .58 |
| ED061302 | 2 | 70 | .44 | .42 | .97 | 1.03 |
| ED061303 | 2 | 51 | .07 | .06 | .76 | .73 |
| ED061304 | 1 | 63 | .26 | .28 | .75 | .68 |
| ED061305 | 2 | 31 | .33 | .29 | .79 | .78 |
| ED061306 | 1 | 72 | .09 | .02 | .75 | .69 |
| ED061307 | 1 | 61 | .35 | .25 | .61 | .55 |
| ED061308 | 2 | 50 | .40 | .28 | .63 | .63 |
| ED061310 | 2 | 57 | .48 | .49 | .72 | .72 |
| ED061313 | 2 | 48 | .41 | .42 | .79 | .78 |
| ED061314 | 2 | 50 | .38 | .33 | .72 | .66 |
| ED061317 | 1 | 39 | .06 | .12 | .83 | .86 |
| ED061317 | 1 | 38 | .16 | .24 | .78 | .76 |
| ED061318 | 2 | 66 | .32 | .30 | .87 | .86 |
| ED061319 | 3 | 54 | .39 | .38 | .60 | .55 |
| ED061320 | 1 | 57 | .23 | .14 | .81 | .74 |
| ED061320 | 1 | 37 | .15 | .06 | .64 | .61 |
| ED061321 | 1 | 74 | -.09 | -.05 | .79 | .74 |
| ED061321 | 1 | 57 | .34 | .41 | .80 | .76 |
| ED061321 | 1 | 94 | .08 | .12 | .89 | .82 |
| ED061322 | 2 | 63 | .22 | .20 | .74 | .78 |
| ED061323 | 1 | 52 | .05 | .05 | .82 | .79 |
| ED061324 | 1 | 87 | .26 | .22 | .76 | .70 |
| ED061326 | 1 | 61 | .11 | .15 | .82 | .80 |
| ED061326 | 1 | 83 | .18 | .18 | .83 | .76 |
| ED061326 | 1 | 57 | -.09 | -.09 | .77 | .73 |
| ED061327 | 1 | 141 | .18 | .15 | .96 | .95 |
| ED061327 | 1 | 82 | .06 | .09 | .92 | .87 |
| ED061327 | 1 | 51 | .15 | .18 | .99 | .95 |
| ED061327 | 1 | 50 | .05 | .04 | .71 | .68 |
| ED061328 | 2 | 50 | .43 | .40 | .80 | .78 |
| ED061328 | 2 | 36 | .37 | .37 | .86 | .85 |
| ED061329 | 2 | 50 | .48 | .50 | .70 | .65 |
| ED061330 | 1 | 52 | .26 | .21 | .77 | .74 |
| ED061332 | 1 | 54 | -.10 | -.04 | .76 | .76 |
| ED061334 | 1 | 54 | .28 | .25 | .66 | .59 |
| ED061335 | 1 | 51 | .12 | .11 | .81 | .80 |
| ED061335 | 1 | 38 | .19 | .19 | .84 | .85 |
| ED061336 | 2 | 60 | .32 | .31 | .84 | .84 |
| ED061336 | 2 | 51 | .35 | .43 | .64 | .64 |
| ED061337 | 2 | 52 | .12 | .11 | .71 | .66 |
| ED061338 | 1 | 50 | .28 | .26 | .75 | .73 |
| ED061339 | 1 | 51 | .16 | .18 | .81 | .79 |
| ED061340 | 1 | 55 | .60 | .48 | .78 | .73 |
| ED061341 | 1 | 40 | .21 | .15 | .97 | .91 |
| ED061342 | 2 | 51 | .40 | .36 | .80 | .83 |
| ED062404 | 2 | 53 | .40 | .34 | .64 | .64 |
| ED062405 | 2 | 52 | .39 | .19 | .64 | .66 |
| ED062406 | 2 | 50 | .16 | .19 | .76 | .73 |
| ED062407 | 1 | 58 | .52 | .50 | .77 | .76 |
| ED062408 | 2 | 59 | .27 | .25 | .83 | .85 |
| ED062410 | 1 | 51 | .18 | .20 | .81 | .76 |
| ED062411 | 1 | 54 | .22 | .18 | .74 | .67 |
| ED062412 | 1 | 50 | -.18 | -.07 | .65 | .59 |
| ED062413 | 1 | 60 | .33 | .32 | .85 | .87 |
| ED062417 | 2 | 80 | .33 | .33 | .82 | .78 |
| ED062418 | 2 | 59 | .19 | .23 | .74 | .70 |
| ED062419 | 1 | 59 | -.06 | -.01 | .71 | .68 |
| ED062420 | 2 | 113 | .15 | .18 | .71 | .66 |
| ED062421 | 2 | 103 | .34 | .33 | .81 | .81 |
| ED062423 | 1 | 113 | .28 | .28 | .75 | .68 |
| ED062424 | 1 | 70 | .29 | .29 | .75 | .72 |
| ED062427 | 1 | 52 | .31 | .32 | .76 | .66 |
| ED062428 | 1 | 50 | .18 | .24 | .74 | .65 |
| ED062429 | 1 | 77 | .36 | .35 | .76 | .74 |
| ED062430 | 1 | 50 | .32 | .27 | .75 | .71 |
| ED062431 | 2 | 53 | .09 | .15 | .60 | .58 |
| ED062432 | 2 | 53 | .24 | .19 | .80 | .78 |
| ED062433 | 2 | 51 | .21 | .27 | .65 | .61 |
| ED062434 | 2 | 56 | .23 | .16 | .72 | .70 |
| ED062435 | 2 | 50 | .42 | .40 | .79 | .78 |
| ED062436 | 2 | 63 | .34 | .33 | .64 | .54 |
| ED062437 | 1 | 50 | .16 | .15 | .74 | .70 |
| ED062438 | 3 | 106 | .15 | .20 | .65 | .68 |
| ED062439 | 2 | 50 | .28 | .25 | .85 | .90 |
| ED062440 | 2 | 55 | .24 | .23 | .76 | .78 |
| ED062440 | 2 | 40 | .28 | .19 | .72 | .73 |
| ED062441 | 1 | 51 | .17 | .19 | .68 | .63 |
| ED062442 | 1 | 70 | .08 | .10 | .78 | .85 |
| ED062443 | 1 | 48 | .49 | .50 | .68 | .62 |
| ED062443 | 1 | 54 | -.09 | -.04 | .62 | .61 |
| ED062443 | 1 | 91 | .28 | .34 | .68 | .65 |
| ED062444 | 2 | 51 | .16 | .08 | .62 | .56 |
| ED062445 | 2 | 50 | .44 | .46 | .70 | .63 |
| ED062447 | 2 | 53 | .20 | .24 | .81 | .79 |
| ED062447 | 2 | 47 | .25 | .23 | .79 | .79 |
| ED062448 | 1 | 75 | .26 | .22 | .72 | .70 |
| ED063380 | 1 | 52 | -.19 | -.10 | .60 | .55 |
| ED063381 | 2 | 72 | .08 | .12 | .75 | .66 |
| ED063382 | 2 | 110 | .19 | .19 | .73 | .69 |
| ED063383 | 2 | 52 | .14 | .13 | .77 | .75 |
| ED063384 | 2 | 52 | .09 | .06 | .92 | .89 |
| ED063385 | 2 | 53 | .39 | .43 | .74 | .73 |
| ED063386 | 2 | 55 | .42 | .39 | .74 | .74 |
| ED063387 | 2 | 50 | .10 | .10 | .71 | .68 |
| ED063388 | 2 | 31 | .57 | .61 | .85 | .91 |
| ED063390 | 3 | 87 | .37 | .39 | .76 | .73 |
| ED063391 | 3 | 59 | .13 | .04 | .57 | .50 |
| ED063392 | 2 | 55 | .23 | .19 | .76 | .74 |
| ED063393 | 1 | 50 | .37 | .31 | .82 | .81 |
| ED063394 | 2 | 51 | .52 | .51 | .82 | .79 |
| ED063395 | 3 | 65 | .30 | .27 | .76 | .75 |
| ED063395 | 3 | 34 | .21 | .17 | .75 | .64 |
| ED063395 | 3 | 6 | .24 | .21 | .79 | .81 |
| ED063396 | 2 | 113 | .32 | .29 | .73 | .73 |
| ED063398 | 2 | 63 | .73 | .65 | .72 | .71 |
| ED063398 | 2 | 124 | .35 | .29 | .75 | .76 |
| ED063400 | 2 | 50 | .29 | .34 | .79 | .74 |
| ED063401 | 2 | 52 | .41 | .40 | .90 | .88 |
| ED063402 | 1 | 51 | .25 | .28 | .86 | .83 |
| ED063403 | 1 | 56 | .36 | .40 | .88 | .88 |
| ED063403 | 1 | 35 | .05 | .12 | .79 | .68 |
| ED063404 | 1 | 60 | .14 | .12 | .64 | .55 |
| ED063405 | 1 | 65 | .34 | .32 | .73 | .72 |
| ED063406 | 2 | 57 | .19 | .19 | 1.01 | .97 |
| ED063407 | 2 | 66 | .18 | .13 | .78 | .74 |
| ED063408 | 2 | 55 | .29 | .26 | .64 | .64 |
| ED063409 | 2 | 53 | .19 | .13 | .68 | .66 |
| ED063410 | 3 | 61 | .56 | .52 | .68 | .64 |
| ED063411 | 1 | 50 | .10 | .08 | .67 | .57 |
| ED063412 | 1 | 50 | .31 | .22 | .77 | .74 |
| ED063413 | 2 | 74 | .22 | .23 | .76 | .69 |
| ED063414 | 2 | 52 | .13 | .12 | .74 | .73 |
| ED063415 | 2 | 50 | .37 | .34 | .77 | .74 |
| ED063416 | 2 | 55 | .09 | .06 | .84 | .80 |
| ED063417 | 1 | 50 | -.07 | -.13 | .74 | .67 |
| ED063418 | 2 | 62 | .26 | .16 | .89 | .89 |
| ED063419 | 2 | 51 | .35 | .34 | .71 | .68 |
| ED065068 | 2 | 81 | .26 | .26 | .71 | .73 |
| ED065560 | 2 | 166 | .08 | .05 | .55 | .51 |
| ED065561 | 2 | 49 | .45 | .47 | .81 | .74 |
| ED065563 | 2 | 50 | .14 | .13 | .72 | .70 |
| ED065566 | 1 | 53 | .02 | .03 | .71 | .65 |
| ED065567 | 3 | 80 | .06 | .14 | .62 | .60 |
| ED065568 | 1 | 56 | .21 | .18 | .88 | .76 |
| ED065569 | 1 | 51 | .22 | .18 | .96 | .94 |
| ED065570 | 2 | 50 | .24 | .23 | .89 | .84 |
| ED065571 | 1 | 64 | .26 | .19 | .78 | .81 |
| ED065572 | 1 | 48 | .20 | .24 | .77 | .71 |
| ED065573 | 2 | 52 | .30 | .25 | .66 | .65 |
| ED065574 | 2 | 52 | .12 | .08 | .83 | .85 |
| ED065575 | 3 | 50 | .62 | .64 | .60 | .55 |
| ED065577 | 2 | 50 | .12 | .06 | .75 | .70 |
| ED065579 | 1 | 83 | .12 | .16 | .65 | .67 |
| ED065580 | 3 | 152 | .13 | .20 | .61 | .57 |
| ED065581 | 1 | 54 | .73 | .76 | .89 | .93 |
| ED065582 | 1 | 50 | .14 | .10 | .59 | .54 |
| ED065583 | 2 | 50 | -.12 | .02 | .67 | .65 |
| ED065606 | 1 | 75 | .08 | .12 | .68 | .66 |
| ED065607 | 1 | 55 | .22 | .22 | .78 | .71 |
| ED065609 | 3 | 80 | .33 | .32 | .62 | .65 |
| ED065610 | 1 | 51 | .05 | .06 | .84 | .83 |
| ED065612 | 2 | 232 | .46 | .44 | .68 | .65 |
| ED065612 | 2 | 60 | .49 | .45 | .70 | .67 |
| ED065613 | 2 | 127 | .15 | .13 | .84 | .84 |
| ED065614 | 1 | 53 | .24 | .23 | .81 | .76 |
| ED065615 | 1 | 61 | -.15 | -.10 | .78 | .70 |
| ED065616 | 2 | 94 | .26 | .22 | .58 | .51 |
| ED065617 | 3 | 71 | .40 | .43 | .72 | .66 |
| ED065618 | 2 | 65 | .25 | .26 | .70 | .66 |
| ED065619 | 2 | 77 | .09 | .13 | .74 | .70 |
| ED065620 | 1 | 100 | .04 | .02 | 1.18 | .69 |
| ED065621 | 2 | 87 | .17 | .16 | .86 | .84 |
| ED065622 | 2 | 41 | .42 | .40 | .83 | .83 |
| ED065622 | 2 | 55 | .31 | .32 | .82 | .82 |
| ED065623 | 2 | 57 | .24 | .11 | .48 | .41 |
| ED065624 | 2 | 59 | .51 | .53 | .72 | .71 |
| ED065625 | 2 | 59 | .44 | .50 | .85 | .89 |
| ED065627 | 1 | 53 | .36 | .37 | .74 | .73 |
| ED065628 | 1 | 199 | .23 | .23 | .77 | .74 |
| ED065631 | 2 | 56 | .28 | .19 | .79 | .75 |
| ED065631 | 2 | 54 | .42 | .38 | .88 | .92 |
| ED065631 | 2 | 54 | .25 | .21 | .78 | .83 |
| ED065632 | 2 | 77 | .55 | .51 | .77 | .78 |
| ED065633 | 2 | 241 | .31 | .30 | .87 | .87 |
| ED065634 | 1 | 50 | .31 | .26 | .83 | .79 |
| ED065635 | 1 | 51 | .15 | .17 | .80 | .82 |
| ED065636 | 1 | 75 | .10 | .13 | .69 | .63 |
| ED065637 | 1 | 52 | .09 | .10 | .92 | .87 |
| ED065638 | 1 | 50 | .21 | .06 | .89 | .94 |
| ED065640 | 1 | 53 | .23 | .21 | .93 | .96 |
| ED065641 | 1 | 66 | .02 | .12 | .68 | .66 |
| ED066458 | 1 | 57 | .56 | .56 | .94 | .95 |
| ED066459 | 1 | 183 | .30 | .26 | .79 | .76 |
| ED066460 | 3 | 80 | .40 | .35 | .75 | .75 |
| ED066461 | 3 | 230 | .23 | .25 | - | - |
| ED066461 | 3 | 51 | .09 | .12 | - | - |
| ED066462 | 1 | 77 | .34 | .33 | .90 | .89 |
| ED066463 | 1 | 58 | .28 | .25 | .75 | .71 |
| ED066464 | 1 | 74 | .27 | .32 | .72 | .66 |
| ED066465 | 1 | 48 | .03 | -.02 | .99 | .98 |
| ED066466 | 2 | 50 | .34 | .34 | .78 | .75 |
| ED066467 | 1 | 53 | .21 | .19 | .77 | .72 |
| ED066468 | 1 | 53 | .52 | .50 | .91 | .94 |
| ED066469 | 2 | 58 | .19 | .16 | .81 | .81 |
| ED066470 | 1 | 54 | .17 | .10 | .80 | .79 |
| ED066472 | 2 | 147 | .23 | .20 | .78 | .76 |
| ED066472 | 2 | 57 | .11 | .07 | .83 | .80 |
| ED066473 | 2 | 100 | .04 | .05 | .94 | .94 |
| ED066474 | 2 | 102 | .22 | .16 | .68 | .70 |
| ED066476 | 3 | 102 | .36 | .29 | .69 | .64 |
| ED066476 | 3 | 93 | .37 | .37 | .83 | .83 |
| ED066476 | 3 | 68 | .28 | .23 | .76 | .69 |
| ED066477 | 3 | 55 | .43 | .43 | .71 | .68 |
| ED066478 | 3 | 72 | .27 | .28 | .70 | .64 |
| ED066478 | 3 | 59 | .48 | .46 | .82 | .77 |
| ED066479 | 2 | 58 | .28 | .29 | .76 | .76 |
| ED066481 | 2 | 73 | .23 | .27 | .81 | .81 |
| ED066482 | 2 | 59 | .19 | .22 | .82 | .76 |
| ED066488 | 2 | 61 | .26 | .23 | .83 | .86 |
| ED066491 | 2 | 198 | .26 | .26 | .75 | .72 |
| ED066491 | 2 | 103 | .33 | .33 | .76 | .72 |
| ED066510 | 2 | 50 | .03 | .02 | .67 | .57 |
| ED066511 | 2 | 79 | .41 | .32 | .67 | .67 |
| ED066512 | 1 | 61 | .15 | .13 | .61 | .55 |
| ED066515 | 2 | 52 | .03 | .03 | .78 | .78 |
| ED066516 | 2 | 56 | .33 | .31 | 1.00 | 1.03 |
| ED066518 | 2 | 75 | .39 | .42 | .80 | .80 |
| ED066519 | 2 | 48 | .15 | .11 | .78 | .78 |
| ED067408 | 1 | 60 | .11 | .11 | .75 | .73 |
| ED067410 | 3 | 51 | .34 | .32 | .79 | .78 |
| ED067410 | 3 | 44 | .52 | .53 | .77 | .78 |
| ED067411 | 1 | 70 | .04 | .11 | .76 | .79 |
| ED067412 | 1 | 58 | .27 | .27 | .79 | .81 |
| ED067413 | 2 | 57 | .06 | .14 | .79 | .79 |
| ED067414 | 2 | 78 | .25 | .25 | .74 | .71 |
| ED067415 | 1 | 50 | .29 | .34 | .77 | .78 |
| ED067416 | 2 | 78 | .27 | .25 | 1.09 | 1.07 |
| ED067417 | 2 | 55 | .15 | .17 | .84 | .85 |
| ED067418 | 2 | 49 | -.03 | -.04 | .80 | .78 |
| ED067420 | 1 | 54 | .15 | .07 | .77 | .78 |
| ED067421 | 2 | 52 | .36 | .35 | .87 | .88 |
| ED067422 | 2 | 63 | .34 | .34 | .85 | .75 |
| ED068539 | 2 | 53 | .49 | .52 | .84 | .82 |
| ED068540 | 1 | 50 | .27 | .36 | .91 | .86 |
| ED068542 | 1 | 58 | .30 | .32 | .83 | .81 |
| ED068543 | 1 | 55 | .51 | .42 | .67 | .64 |
| ED068545 | 1 | 55 | .28 | .21 | .73 | .69 |
| ED068548 | 2 | 60 | .02 | .02 | .71 | .64 |
| ED068549 | 1 | 66 | .08 | .14 | .78 | .79 |
| ED068550 | 2 | 67 | .11 | .10 | .71 | .69 |
| ED068551 | 3 | 59 | .22 | .24 | .78 | .72 |
| ED068553 | 3 | 55 | .31 | .29 | .75 | .75 |
| ED068555 | 1 | 57 | .15 | .11 | .74 | .80 |
| ED068556 | 3 | 76 | .11 | .16 | .85 | .87 |
| ED068558 | 1 | 52 | .13 | .08 | .79 | .78 |
| ED068559 | 2 | 84 | .53 | .49 | .81 | .81 |
| ED068560 | 1 | 87 | .27 | .19 | .67 | .64 |
| ED068561 | 1 | 100 | .39 | .41 | .80 | .75 |
| ED068562 | 3 | 57 | .27 | .27 | .77 | .69 |
| ED068563 | 2 | 50 | .13 | .10 | .91 | .84 |
| ED068564 | 2 | 76 | .23 | .24 | .67 | .63 |
| ED068565 | 1 | 85 | .11 | .08 | .67 | .64 |
| ED068568 | 1 | 56 | .22 | .26 | .72 | .70 |
| ED069541 | 2 | 64 | .22 | .24 | .93 | .93 |
| ED069626 | 2 | 55 | .26 | .19 | .76 | .75 |
| ED069628 | 1 | 49 | .09 | .08 | .82 | .79 |
| ED069629 | 1 | 51 | .36 | .33 | .83 | .78 |
| ED069630 | 1 | 50 | .02 | .05 | .88 | .86 |
| ED069631 | 2 | 59 | .31 | .31 | .95 | .92 |
| ED069632 | 2 | 55 | .30 | .28 | .71 | .70 |
| ED069633 | 3 | 64 | .05 | .08 | .62 | .61 |
| ED069634 | 1 | 59 | .30 | .25 | .75 | .70 |
| ED069634 | 1 | 56 | -.03 | -.03 | .71 | .67 |
| ED069638 | 3 | 53 | .23 | .21 | .56 | .58 |
| ED069639 | 1 | 61 | .19 | .15 | .79 | .80 |
| ED069640 | 3 | 53 | .39 | .32 | .65 | .65 |
| ED069642 | 2 | 32 | .20 | .21 | .81 | .75 |
| ED069643 | 1 | 60 | .37 | .31 | .80 | .88 |
| ED069643 | 1 | 56 | .02 | .16 | .61 | .55 |
| ED069644 | 1 | 50 | .22 | .17 | .74 | .71 |
| ED069646 | 1 | 69 | -.09 | -.04 | .75 | .73 |
| ED069647 | 2 | 74 | .20 | .19 | .76 | .80 |
| ED069648 | 2 | 55 | .02 | .00 | .57 | .56 |
| ED069649 | 2 | 72 | .43 | .35 | .73 | .72 |
| ED069650 | 2 | 53 | .26 | .22 | .66 | .65 |
| ED069651 | 2 | 50 | .27 | .23 | .60 | .56 |
| ED069652 | 2 | 50 | .45 | .42 | .83 | .81 |
| ED069764 | 1 | 70 | .16 | .10 | .83 | .85 |
| ED069765 | 2 | 66 | .19 | .17 | .59 | .64 |
| ED069766 | 1 | 75 | .22 | .19 | .88 | .88 |
| ED069767 | 1 | 69 | .17 | .16 | .72 | .73 |
| ED069769 | 1 | 136 | .10 | .09 | .98 | .95 |
| ED069770 | 2 | 64 | .31 | .31 | .84 | .86 |
| ED069774 | 2 | 50 | .25 | .32 | .92 | .97 |
| ED069775 | 2 | 50 | .20 | .23 | .72 | .67 |
| ED069776 | 2 | 54 | .17 | .15 | .77 | .72 |
| ED069777 | 2 | 61 | .37 | .37 | .75 | .76 |
| ED069778 | 2 | 52 | .27 | .27 | .79 | .81 |
| ED069779 | 2 | 84 | .17 | .19 | .73 | .71 |
| ED069780 | 1 | 53 | .40 | .34 | .79 | .79 |
| ED070771 | 1 | 57 | .22 | .23 | .75 | .69 |
| ED070772 | 1 | 53 | .17 | .18 | .80 | .79 |
| ED070773 | 3 | 62 | .52 | .57 | .89 | .82 |
| ED070774 | 2 | 120 | .35 | .31 | .71 | .68 |
| ED070775 | 1 | 55 | .29 | .32 | .76 | .80 |
| ED070776 | 2 | 61 | .45 | .41 | .72 | .69 |
| ED072046 | 1 | 92 | .23 | .24 | .90 | .91 |
| ED072046 | 3 | 122 | .21 | .21 | .67 | .63 |
| ED072049 | 1 | 53 | .20 | .15 | .81 | .81 |
| ED072050 | 3 | 82 | .20 | .20 | .75 | .70 |
| ED072051 | 1 | 51 | .18 | .24 | .63 | .61 |
| ED072052 | 2 | 50 | .28 | .20 | .68 | .68 |
| ED072052 | 2 | 49 | .48 | .48 | .82 | .88 |
| ED072052 | 2 | 70 | .24 | .23 | .82 | .82 |
| ED072055 | 3 | 84 | .14 | .18 | .64 | .57 |
| ED072055 | 3 | 75 | .32 | .28 | .63 | .61 |
| ED072056 | 1 | 55 | .27 | .19 | .66 | .65 |
| ED072056 | 2 | 80 | .41 | .34 | .60 | .55 |
| ED072056 | 2 | 53 | .75 | .64 | .65 | .57 |
| ED072056 | 2 | 61 | .47 | .50 | .73 | .69 |
| ED072056 | 2 | 71 | .02 | -.03 | .60 | .57 |
| ED072056 | 2 | 73 | .04 | .03 | .56 | .53 |
| ED072056 | 2 | 54 | .35 | .37 | .56 | .56 |
| ED072056 | 2 | 70 | .08 | .10 | .67 | .62 |
| ED072056 | 2 | 82 | .40 | .31 | .55 | .50 |
| ED072056 | 2 | 86 | .33 | .29 | .59 | .56 |
| ED072057 | 2 | 50 | .25 | .29 | .87 | .90 |
| ED072058 | 1 | 86 | .18 | .20 | .74 | .74 |
| ED072059 | 1 | 53 | .23 | .27 | .89 | .86 |
| ED072059 | 1 | 60 | .43 | .39 | .85 | .83 |
| ED072060 | 1 | 64 | .18 | .15 | .76 | .72 |
| ED072060 | 1 | 64 | .18 | .15 | .76 | .72 |
| ED072061 | 1 | 51 | .30 | .33 | .82 | .75 |
| ED072062 | 1 | 50 | .30 | .25 | .87 | .86 |
| ED072063 | 1 | 58 | .03 | .04 | .75 | .71 |
| ED072065 | 1 | 53 | .22 | .15 | .58 | .50 |
| ED072066 | 2 | 53 | .32 | .29 | .76 | .72 |
| ED072067 | 2 | 98 | .52 | .53 | .76 | .76 |
| ED072068 | 1 | 66 | -.04 | .03 | .74 | .68 |
| ED072069 | 2 | 106 | .20 | .26 | .61 | .59 |
| ED078070 | 1 | 31 | .33 | .37 | .54 | .49 |
| ED078070 | 1 | 34 | .18 | .19 | .79 | .79 |
| ED100969 | 2 | 81 | .14 | .16 | .68 | .63 |
| ED100969 | 2 | 99 | .21 | .22 | .84 | .83 |
| ED103450 | 1 | 202 | .22 | .22 | .90 | .91 |
| ED103451 | 1 | 348 | .25 | .25 | .75 | .75 |
| ED103456 | 2 | 266 | .29 | .28 | .86 | .88 |
| ED103489 | 2 | 326 | .47 | .44 | .74 | .73 |
| ED103491 | 2 | 407 | .36 | .37 | .78 | .76 |
| ED103492 | 1 | 186 | .32 | .33 | .86 | .88 |
| ED103495 | 2 | 291 | .16 | .20 | .85 | .84 |
| ED117132 | 1 | 78 | .17 | .17 | .85 | .89 |
| ED117159 | 2 | 204 | .19 | .16 | .74 | .73 |
| ED117160 | 2 | 234 | .11 | .08 | .82 | .82 |
| ED118598 | 2 | 243 | .27 | .29 | .87 | .89 |
| ED158039 | 2 | 253 | .18 | .21 | .82 | .68 |
| ED223695 | 2 | 154 | .38 | .36 | .82 | .84 |
| ED223696 | 2 | 404 | .22 | .24 | .82 | .81 |
| ED223707 | 2 | 123 | .16 | .16 | .81 | .75 |
| ED223714 | 2 | 239 | .20 | .23 | .93 | .94 |
| ED223718 | 2 | 201 | .32 | .31 | .75 | .73 |
| ED223722 | 2 | 194 | .23 | .19 | .84 | .86 |
| ED223723 | 2 | 496 | .30 | .30 | .88 | .90 |
| ED224826 | 1 | 644 | .22 | .21 | .75 | .75 |
| ED263137 | 2 | 272 | .18 | .37 | .75 | .80 |
|  |  |  |  |  |  |  |

Note. Job Cplx = job complexity 1 = low level, 2 = medium level, 3= high level; *r_g_ =* observed validity of G; *r_gvn_* = observed validity of GVN; *u_g_* = range restriction value of G; *u_gvn_* = range restriction value of GVN.

Supplementary Material 4. Validity Coefficients and Range Restriction Values of G and GVN Composites for Production Records.

|  |  |  |  |  |  |  |
| --- | --- | --- | --- | --- | --- | --- |
| ERIC ID | Job Cplx | Sample | *r_g_* | *r_gvn_* | *u_gvn_* | *u_g_* |
|  |  |  |  |  |  |  |
| ED060117 | 1 | 58 | .24 | .25 | .72 | .67 |
| ED060117 | 1 | 91 | .11 | .11 | .73 | .67 |
| ED060099 | 1 | 58 | .31 | .36 | .73 | .66 |
| ED061322 | 2 | 63 | .16 | .13 | .78 | .78 |
| ED060096 | 2 | 41 | .15 | .13 | .69 | .65 |
| ED066471 | 2 | 50 | .11 | .11 | .89 | .88 |
| ED065611 | 1 | 51 | .08 | .08 | .98 | 1.01 |
| ED060101 | 2 | 50 | .37 | .03 | .74 | .68 |
| ED060128 | 1 | 50 | .21 | .19 | .79 | .79 |
| ED060081 | 1 | 27 | -.23 | -.18 | .65 | .66 |
| ED060081 | 1 | 24 | -.18 | -.17 | .69 | .66 |
| ED060081 | 1 | 52 | -.16 | -.12 | .77 | .74 |
| ED061317 | 1 | 50 | .35 | .37 | .78 | .74 |
| ED061317 | 1 | 38 | .16 | .22 | .81 | .76 |
| ED060072 | 1 | 31 | .32 | .38 | .90 | .90 |
| ED061321 | 1 | 44 | -.02 | -.06 | .71 | .65 |
| ED059287 | 1 | 100 | .03 | .04 | - | - |
| ED059287 | 1 | 65 | .08 | .04 | - | - |
| ED059287 | 1 | 73 | .03 | .05 | .80 | .74 |
| ED059284 | 1 | 27 | .25 | .35 | - | - |
| ED059284 | 1 | 23 | .28 | .31 | - | - |
| ED059284 | 1 | 47 | .16 | .20 | - | - |
| ED059284 | 1 | 36 | .50 | .52 | - | - |
| ED059284 | 1 | 58 | .26 | .23 | .71 | .68 |
| ED059284 | 1 | 75 | -.05 | -.04 | .71 | .65 |
| ED069645 | 1 | 57 | .18 | .20 | .83 | .77 |
| ED059285 | 2 | 60 | .17 | .20 | .70 | .62 |
| ED059285 | 2 | 42 | .18 | .03 | .61 | .56 |
| ED059303 | 1 | 34 | .31 | .29 | .82 | .77 |
| ED061327 | 1 | 53 | -.01 | .07 | .71 | .66 |
| ED969771 | 2 | 51 | .33 | .29 | .69 | .60 |
|  |  |  |  |  |  |  |

Note. Job Cplx= job complexity 1 = low level, 2 = medium level, 3 = high level; *r_g_ =* observed validity of G; *r_gvn_* = observed validity of GVN; *u_g_* = range restriction value of G; *u_gvn_* = range restriction value of GVN.

Supplementary Material 5. Validity Coefficients and Range Restriction Values of G and GVN Composites for Work Sample Tests.

|  |  |  |  |  |  |  |
| --- | --- | --- | --- | --- | --- | --- |
| ERIC ID | Job Cplx | Sample | *r_g_* | *r_gvn_* | *u_gvn_* | *u_g_* |
|  |  |  |  |  |  |  |
| ED063391 | 1 | 57 | .08 | .08 | .69 | .65 |
| ED062426 | 1 | 80 | .37 | .43 | .79 | .75 |
| ED061313 | 2 | 57 | .20 | .21 | .77 | .77 |
| ED066491 | 2 | 89 | .35 | .34 | .74 | .61 |
| ED059290 | 2 | 66 | .37 | - | - | .65 |
| ED063397 | 2 | 49 | .02 | .03 | .77 | .80 |
| ED059288 | 2 | 130 | .29 | .34 | .68 | .68 |
| ED059288 | 2 | 60 | .41 | .46 | .54 | .51 |
| ED059288 | 2 | 50 | .45 | .48 | .54 | .49 |
| ED059288 | 2 | 58 | .50 | .55 | .81 | .82 |
| ED065612 | 2 | 63 | .30 | .18 | .56 | .48 |
|  |  |  |  |  |  |  |

Note. Job Cplx= job complexity 1= low level, 2 = medium level, 3 = high level; *r_g_ =* observed validity of G; *r_gvn_* = observed validity of GVN; *u_g_* = range restriction value of G; *u_gvn_* = range restriction value of GVN.
